# Electrical stimulation in upper limb assistance: opportunities and challenges

**DOI:** 10.3389/fnins.2025.1702889

**Published:** 2026-01-16

**Authors:** Nathan Routledge, Dingguo Zhang, Benjamin Metcalfe

**Affiliations:** 1 Department of Electronic and Electrical Engineering, University of Bath, Bath, United Kingdom; 2 Bath Institute for the Augmented Human, University of Bath, Bath, United Kingdom; 3 Centre for Bioengineering and Biomedical Technologies, University of Bath, Bath, United Kingdom

**Keywords:** assistive technology (AT), electrical stimulation (ES), hybrid assistive devices, nerve stimulation, neuromuscular stimulation, neuroprosthesis, upper limb (UL)

## Abstract

The global rise in non-communicable diseases, alongside an aging population, is expected to increase the prevalence of motor impairments and, therefore, the need for assistive care. Upper limb impairments can significantly affect independent living and increase long-term care costs. Wearable assistive devices incorporating electrical stimulation (ES) offer a promising solution to support independence and help alleviate pressures on both formal and informal care provision. The development of hybrid systems, which integrate aspects of robotics and electrical stimulation, aim to overcome the limitations associated with single-modality devices. However, there is limited information on the most appropriate electrical stimulation protocols to use, or on what challenges may be faced in doing so. Correspondingly, this narrative review addresses this gap through assessing the role of electrical stimulation in upper limb assistive technology. By evaluating user requirements and identifying challenges with current stimulation strategies, this review highlights the potential benefits of exploring alternative protocols, beyond conventional functional electrical stimulation (FES) techniques, for upper limb assistance. In particular, addressing practical difficulties of stimulation is likely to be critical for successful user uptake and minimizing device abandonment. The paper subsequently reviews several stimulation strategies which may offer novel research directions and opportunities in the development of upper limb assistive technologies.

## 1 Introduction

### 1.1 Scope and impact

Human hands are a complex biological tool, used not only for completing functional tasks, but also for human expression and connection. Accordingly, the loss of upper limb (UL) function is among the most significant and devastating impairments a person may experience (Harnett et al., 2020). While various conditions can lead to UL motor dysfunction, the chronic effects of a stroke remain a leading cause of adult disability worldwide (Pollock et al., 2014), where hemiparesis including UL impairment is a common outcome (Hatem et al., 2016). Globally, in 2021, there were over 89 million people living with the effects of a stroke, and approximately 12 million new cases (Feigin et al., 2025). Of those affected, generally nearly two-thirds leave hospital with a disability, with more than 75% reporting arm weakness that limits their independence and ability to perform activities of daily living (ADLs) (Lawrence et al., 2001; Camona et al., 2018). Consequently, stroke care amounts to an estimated global cost of $890 billion, USD (Feigin et al., 2025), with reports indicating approximately 60% of this is associated with the expenses of informal care, likely due to difficulties with performing self-care and ADLs independently (Patel et al., 2020).

**Figure 1:**
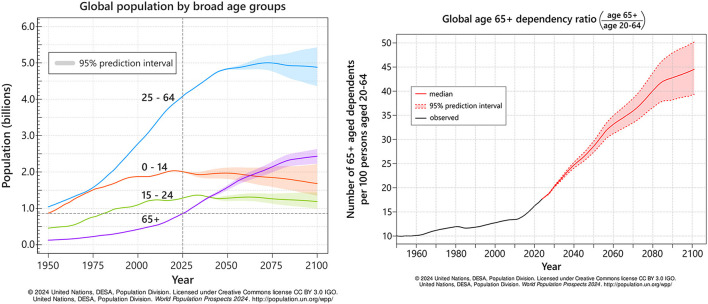
Observed and predicted changes to the global population from 1950 to 2100 (United Nations, Department of Economic and Social Affairs, Population Division, 2024). *Graph appearance adjusted from original source*. **(A)** Changes to global population by broad age groups. There is a substantial predicted increase in adults aged 65+, close to doubling in the next 25 years. **(B)** Changes to the global 65+ dependency ratio; a higher ratio suggests an increased pressure on social and informal care.

These figures are expected to grow (Feigin et al., 2025). By 2030, a predicted one in six people globally will be aged 60 or older (World Health Organization, 2021); significant as the burden of stroke-related disablement is greatest in those aged 60–84 (Feigin et al., 2020). In combination with expected increases in people with a disability, partly due to global population aging (Figure 1A), but also from the rising rates of non-communicable diseases, such as stroke (World Health Organization, 2011; Feigin et al., 2020), this suggests an increasing demand for effective solutions to chronic motor dysfunction. Simultaneously, a predicted leveling or decreasing of younger population groups may exacerbate the pressures on both formal and informal, unpaid care; as reflected in the growing dependency ratio (Figure 1B).

In addition to stroke, spinal cord injury (SCI) is another major cause of UL motor impairment. In 2019, over 20 million people worldwide were living with an SCI, and there was an annual incidence of approximately 900,000 people who sustained a new injury (Safdarian et al., 2023). Of these, cervical-level injuries account for a slight majority of cases, estimated around 50%–54% (Stephan et al., 2015; Harnett et al., 2020; Safdarian et al., 2023). As injury at the cervical vertebrae level, can cause motor dysfunction in both upper and lower limbs, independent living is significantly affected. This not only impacts UL-related ADLs, but can also severely restrict an individual's general mobility, as mobility aids often require UL use (Harnett et al., 2020). It, therefore, corresponds that the restoring UL function is considered a high priority by individuals with an SCI (Anderson, 2004; Harnett et al., 2020).

Given the growing prevalence of motor impairments from stroke, SCI, as well as other neuromuscular conditions, combined with the increasing demand on care systems, there is a clear need for practical and effective solutions. Technologies that can provide assistance with UL motion and functionality, aid independent living across a range of ADLs, and which may offer rehabilitative potential for relevant users, represent a promising option to improve quality of life, reduce care pressures, and address critical gaps in long-term disability support.

### 1.2 Electrical stimulation for upper limb assistance

**Figure 2:**
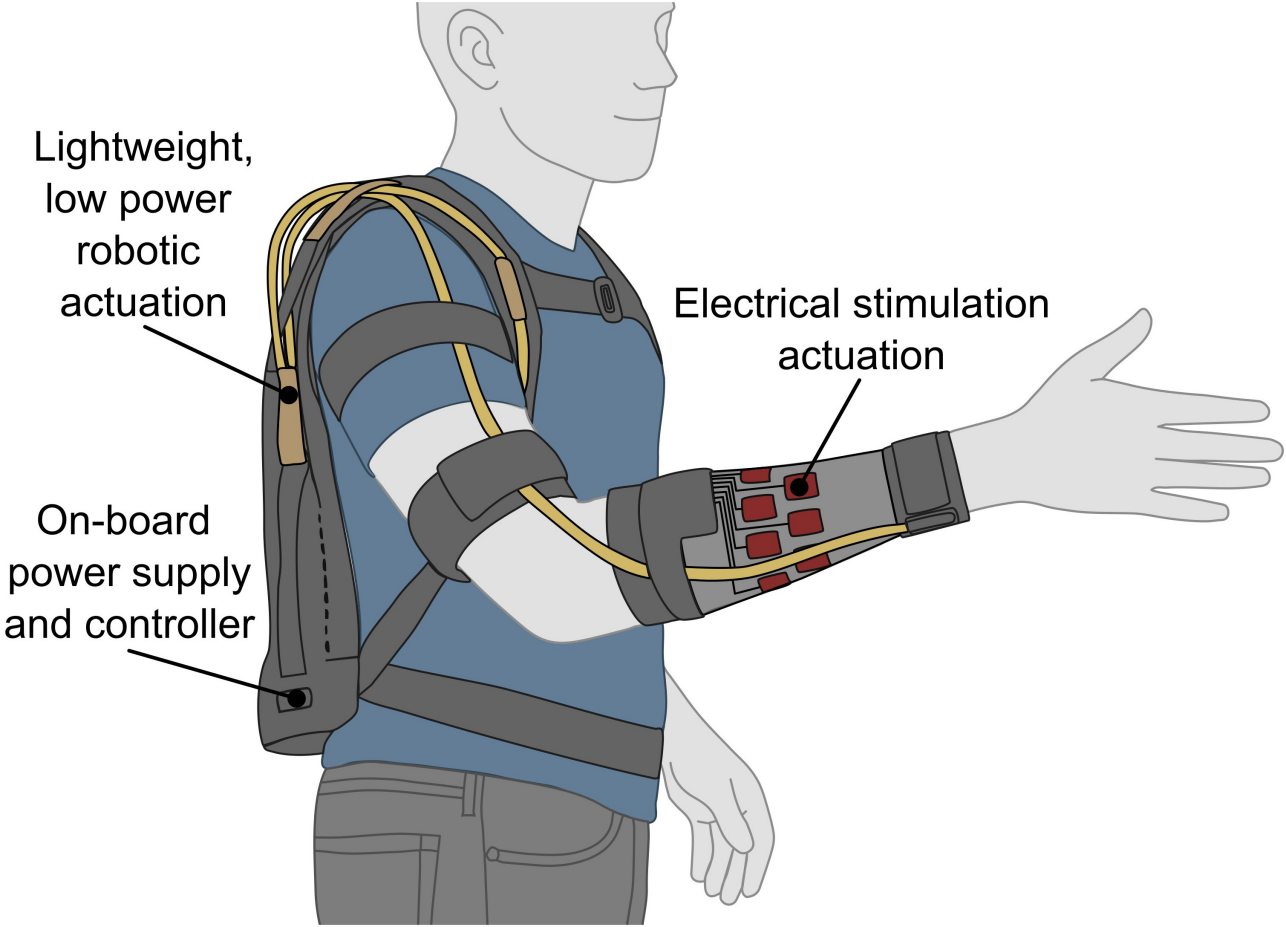
Concept drawing of a wearable, hybrid upper limb assistive device, or neuroprosthetic, incorporating robotics and electrical stimulation as actuation methods.

With the developments in rehabilitation robotics over the last two decades, a technological-based method for achieving functional motion assistance is considered an important possible aid, both directly for those with UL motor impairments, and indirectly in easing pressures on care settings (Gull et al., 2020; Gandolla et al., 2020). Alongside this, the use of electrical stimulation (ES) to prompt muscle contraction, can subsequently elicit joint movements and may also provide therapeutic benefits to some people with a neurological injury, or forms of paresis (Wang et al., 2020). Hence ES may be used on its own, or jointly with robotics or a mechanical orthosis, forming a “hybrid” device or neuroprosthesis, to assist with generating UL functional motion. An example concept device is illustrated in Figure 2.

Reports suggest that incorporating ES in a hybrid or neuroprosthetic device can result in a more power efficient system than robotic actuation alone by making use of the user's muscles as “biological actuators” (Wolf et al., 2017); thereby bringing advantages of greater wearability, portability, acceptance and usability (Wang et al., 2020). However, challenges remain in the use of assistive electrical stimulation for UL applications. The precise, selective and functional control of stimulation-elicited motion is difficult to achieve due to the non-linearities and delays inherently involved with biological muscle actuation (Dunkelberger et al., 2020). Furthermore, long-term use and high intensity stimulation can be limited by muscle fatigue and may be painful in some people with intact sensation (Stewart et al., 2017).

Nevertheless, a recent review examining the efficacy of using hybrid UL neuroprostheses—which incorporate ES—following a stroke, highlights the growth of the field with reports of 20 of the 32 reviewed hybrid systems being published after 2014 (Höhler et al., 2024). Significantly, the meta-analysis on the efficacy results from seven randomized control trials (RCTs) demonstrated a positive restorative effect of hybrid devices on UL motor recovery after a stroke, with improvements demonstrated in both sub-acute and chronic phases (Höhler et al., 2024). Similar positive effects of UL assistance neuroprostheses have been reported in people with an SCI (Readioff et al., 2021), indicating the assistive and rehabilitative potential of UL hybrid devices across several clinical populations.

### 1.3 Summary and aim

Existing reviews provide details of research-based and early commercial UL assistive neuroprosthetic devices in their ability to assist or rehabilitate UL function (Höhler et al., 2024; Hoppe-Ludwig et al., 2021; Readioff et al., 2021; Dunkelberger et al., 2020; Stewart et al., 2017; Resquín et al., 2016), details of the available neurotechnologies for restoring UL function (Losanno et al., 2023; Harnett et al., 2020; Marquez-Chin and Popovic, 2020; Hatem et al., 2016) and descriptions of user requirements for general UL rehabilitative and assistive devices (Kobbelgaard et al., 2021; Boser et al., 2020; Waddell et al., 2016).

For current UL hybrid and neuroprosthetic assistive systems, much of the research has focused on the development of the physical design, actuation and control techniques, while typically employing conventional methods of functional electrical stimulation (FES) (Malešević et al., 2012; Pedrocchi et al., 2013; De Marchis et al., 2016; Wang et al., 2020; Ambrosini et al., 2021). However, challenges remain in using conventional FES with UL applications, particularly in achieving consistent and fatigue-resistant motions over extended use throughout a day. These challenges largely stem from the compact physiology, and synergistic nature of UL musculature compared to the lower limbs. Moreover, while reviews on the efficacy of UL FES highlight positive rehabilitative effects, often the evidence quality is mixed or low (Eraifej et al., 2017; Khan et al., 2023). Therefore, while study outcomes are promising, this limits the strength of the conclusions. As such, the FES paradigms typically employed in hybrid systems may not be fully optimized, and particularly for use in UL assistance.

Beyond conventional FES, a variety of alternative electrical stimulation paradigms exist that can also elicit and support joint motion. These include techniques that stimulate sensorimotor nerves or spinal roots, thereby engaging slightly differing mechanisms of action, and potentially offering distinct benefits over conventional FES protocols. However, to the authors' knowledge, currently no critical evaluation exists that assesses different ES techniques in their suitability for UL assistive applications.

Correspondingly, this paper addresses that gap, synthesizing details from reviews of user requirements with a critical analysis of current stimulation strategies, and their challenges and limitations in UL applications. Based on this analysis, it is suggested that FES techniques may not be ideally suited for assistive use in the UL. Instead, alternative ES protocols may better address the outstanding challenges that likely hinder the translation and uptake of UL assistive devices beyond research environments. Hence this paper subsequently presents a narrative review of ES paradigms that reportedly elicit UL motor responses, and discusses their potential for further investigation and possible integration into UL assistive technologies.

## 2 Electrical stimulation (ES)

### 2.1 Overview

**Figure 3:**
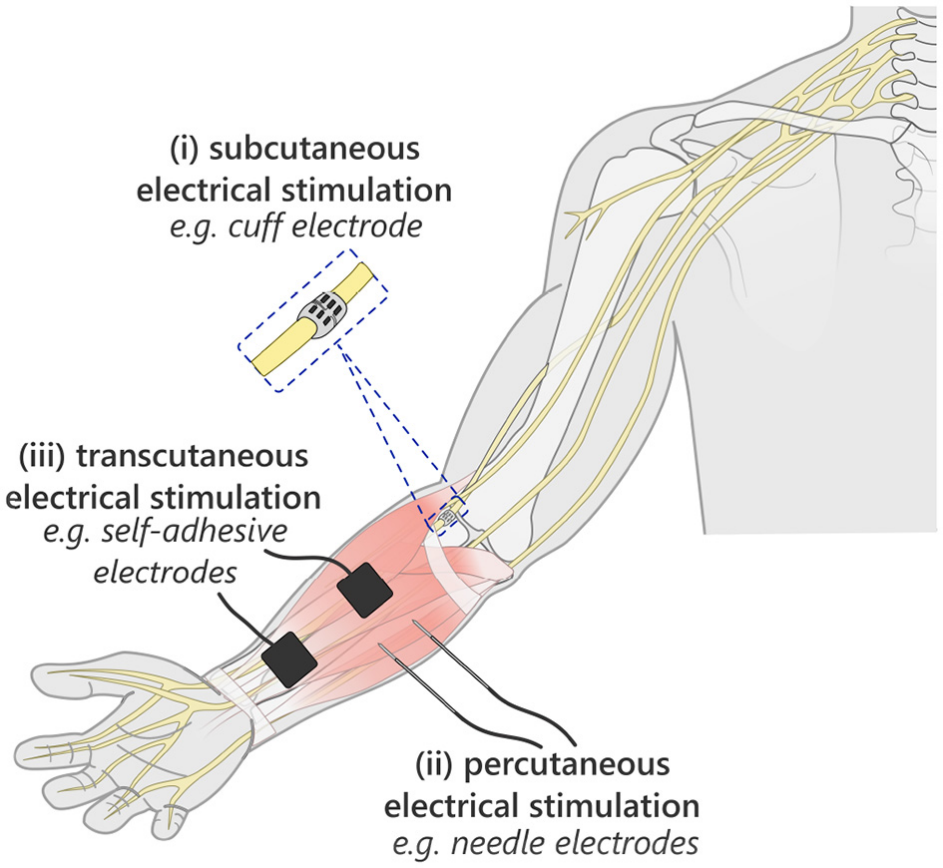
Illustration of typical application method categories for delivering ES, by level of invasiveness. The greater the level of invasiveness the greater the selectivity, and typically lower stimulation intensity required. However, the more invasive techniques come with the risks and challenges of surgery, implanted foreign bodies and infection.

Electrical stimulation applied to neuromuscular tissue has been in clinical use for the treatment and rehabilitation of motor impairments for over 50 years (Bussel, 2015); where early uses were in stimulating the peroneal nerve to correct for foot drop in people with chronic hemiplegic effects from a stroke (Doucet et al., 2012). The three fundamental techniques for delivering ES, being subcutaneous, percutaneous and transcutaneous methods, are illustrated in Figure 3, demonstrating the range of “invasiveness” (Rushton, 1997; Marquez-Chin and Popovic, 2020).

There is an inherent trade-off between the selectivity of the technique and its ease and risk of application. Due to the greater likelihood of permanence with their UL impairment, it has been suggested that individuals with an SCI are more likely to be open to invasive techniques. However, while reviews indicate that some people with an SCI may be open to implanted devices – predominantly to decrease the visibility of an assistive device – many also conflictingly express that requiring surgery to install such a device is unacceptable, and as such, state non-invasiveness as a high priority (Brown-Triolo et al., 2002; Anderson, 2004). The installation of invasive devices may, therefore, be limited to those who require a separate surgical intervention, but in which installation of subcutaneous electrodes may be performed coincidentally. For those where UL therapeutic rehabilitation is targeted, and so may have some chance of recovering function, it is generally accepted that non-invasiveness is a valued requirement for an assistive device. Consequently, there is a demand to target non-invasive methods for use in assistive technology (AT), and accordingly, this paper focuses on transcutaneous stimulation paradigms.

While transcutaneous ES is used clinically for purposes such as improving muscle strength and joint range of motion (RoM) (Doucet et al., 2012), when stimulation is used to produce functional and coordinated limb movements, this is generally termed FES. Conventionally, FES is performed by placing an active electrode on the skin over the belly of the muscle selected for activation, and targeting the muscle motor point. This technique is more specifically termed motor point stimulation (MPS) (Milosevic et al., 2020). Alternatively, electrodes may be placed over a motor nerve trunk located distally from, but which provides innervation to, the target muscle; often termed peripheral nerve stimulation (PNS) (Milosevic et al., 2020; Nakagawa et al., 2023), or functional nerve stimulation (FNS) (Tharu et al., 2023). Consequently, the use of these stimulation techniques necessitate the presence of innervated muscle, thereby having intact and excitable lower motor neurons (LMNs) within the targeted region (Peckham and Knutson, 2005). This limits the use of assistive ES for clinical conditions involving LMN damage. Although in cases of denervated muscles due to LMN lesions, ES can be applied therapeutically for maintaining muscle composition and metabolic conditions, or mitigating complications from prolonged disuse, though this typically requires specialized protocols (Bersch and Fridén, 2021; Mayr and Bersch-Porada, 2024). Nevertheless, ES has been documented for rehabilitative and assistive uses in cases of stroke recovery, SCI, traumatic brain injury (TBI), multiple sclerosis and cerebral palsy (Peckham and Knutson, 2005; Taylor et al., 2013; Eftekhar et al., 2018).

### 2.2 Challenges of FES use for upper limb assistance

In general, any use of ES is inherently inefficient when compared to biological motor behavior. Stimulation induces excessive muscle fatigue, which has predominantly been attributed to eliciting a synchronized and non-physiological muscle fiber recruitment pattern and order (Doucet et al., 2012). For stimulation used within an assistive device, where reliable use is expected over several hours, regulating muscle fatigue is a key challenge to address. Various techniques have been investigated for reducing fatigue: this includes tuning stimulation parameters for a maximally delayed fatigue reaction; the use of variable frequency pulse patterns; techniques for optimizing the electrode position over muscle motor points; control strategies; and the use of spatially and/or temporally distributed stimulation, often via multichannel stimulation (Rushton, 1997; Ibitoye et al., 2016). Research has also suggested that the use of sensorimotor nerve stimulation involving afferent pathways, may generate muscle fiber recruitment in a more biologically typical form, and thereby decrease the rate of muscle fatigue (Shin et al., 2018). However, as yet there is no generally accepted method for improving the fatigue resistance of ES (Ibitoye et al., 2016).

Furthermore, natural, volitional human grasping involves the synergistic motion from a combination of extrinsic and intrinsic, superficial and deep muscles of the hand and forearm (Crema et al., 2018). However, for FES-activated hand grasping patterns, electrodes are typically placed on the anterior and posterior forearm, targeting the motor points of the superficial extrinsic finger and wrist muscles. This gives limited activation of the deeper extrinsic muscles, and often excludes the intrinsic hand and thenar muscles, which may exacerbate fatigue effects.

**Figure 4:**
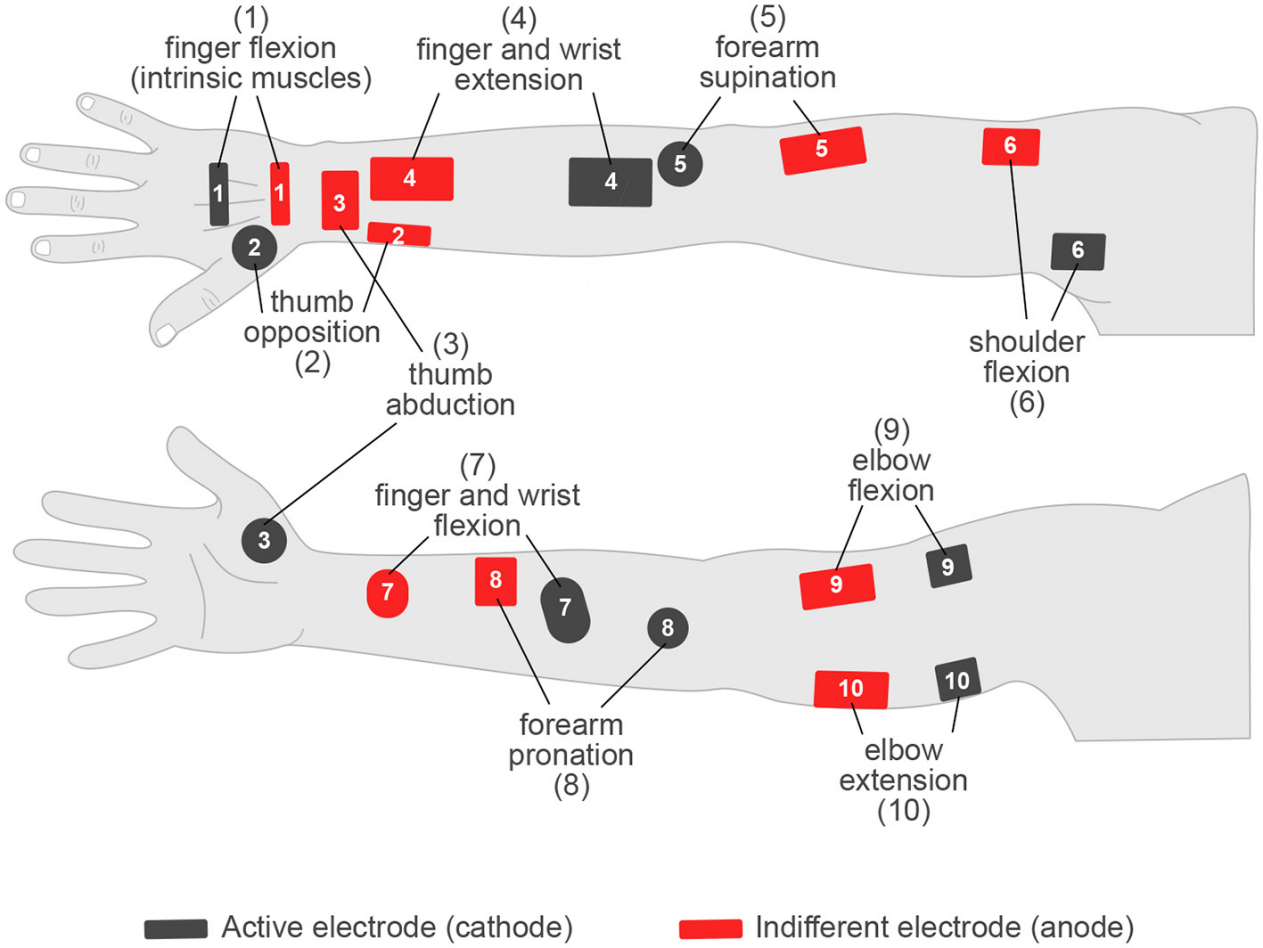
Illustration approximately highlighting the typical electrode locations used in conventional FES of the upper limb (Baker et al., 2000; Humphreys et al., 2011; Axelgaard, 2025). Please note wrist flexion and extension are shown here combined with finger flexion and extension, however wrist motion can also be targeted separately. There are also variations for several motions which are not shown. Additionally, FES for countering shoulder subluxation and for providing shoulder girdle stability is not included.

In addition to this, achieving accurate and selective muscle activation can be troublesome with upper limb FES due to the compact and overlying nature of the extrinsic forearm compartment muscles. Moreover, natural variations in physiology can cause optimal stimulation locations to vary between people. A sub-optimal electrode placement may require higher current amplitudes to achieve the desired effect, or lead to the “overflow” phenomenon, whereby adjacent muscles are undesirably also excited (Koutsou et al., 2016). Typical electrode locations for upper limb FES are illustrated in Figure 4. In some cases, use of the overflow phenomenon may be beneficial to reduce the number of electrodes used to achieve synergistic joint motions, such as that for combined finger and wrist extension, or flexion, as shown by electrodes (4) and (7) respectively. However, as highlighted in Figure 4, the ability to stimulate most UL joint motions typically requires electrodes to be placed over much of the entire upper limb (Niu et al., 2019).

Some people may also experience discomfort with the use of FES. This is most prevalent with high current densities (Doucet et al., 2012), and therefore, provides an added challenge when attempting to selectively target the smaller-scale UL muscles with small electrodes. Though discomfort can also be due to the poor placement of electrodes, thereby requiring higher stimulation currents to elicit the desired effect (Doucet et al., 2012). It corresponds, therefore, that the time and resources taken to locate optimal electrode positions can often be a restrictive factor to the practicalities of using an ES system, both in clinic and during home-use. To counter this, researchers have investigated the use of multi-electrode arrays (MEAs), comprising several small electrodes joined on a non-conductive array. With this, an optimal electrode may be tested and selected, programmatically or otherwise, without repositioning the entire array (Koutsou et al., 2016). Correspondingly, studies have provided stimulation mapping of the forearm to guide the location of MEAs for grasping, and to determine the activated muscles and individual joint motions available (Nathan, 1979; Popović Maneski et al., 2016). Nevertheless, the calibration of such MEAs to optimize the stimulating electrodes used is time-consuming, and often involves additional applied stimulation (Malešević et al., 2010; Usman et al., 2020). This may add to muscle fatigue effects before use of the device for its desired purpose. The development of practical and suitable calibration and configuration methods is then an additional challenge, which would be necessary to successfully translate such MEA techniques beyond the research environment.

Therefore, while FES and rehabilitative ES protocols for the upper limb see some use in clinical settings, as yet there is limited translation from research to clinical or daily-living contexts. Factors such as development cost constraints, undertaking medical device regulatory procedures, and difficulties in enabling clinical adoption of AT provide hurdles in translating devices beyond research settings. However, to enable the regular and consistent use of upper limb ES systems, there also remain some practical challenges of device design and aspects of user requirements to address. These are also important considerations as exploring, addressing and documenting user requirements, and the corresponding device design decisions, is encouraged from the early stages of research to help streamline later regulatory procedures (Crook and Vanhoestenberghe, 2024).

## 3 User requirements

Likely user populations, such as individuals with lasting effects from a stroke or SCI, are considered to be heterogeneous, even among people with the same diagnosis (Smaby et al., 2004; Anwer et al., 2022). Therefore, the creation of a clear and well-defined set of user characteristics and, subsequently, device design specifications is challenging. Equally, clear standards and guidelines for the development of human-robot interactions in wearable devices are also considered lacking (Meyer et al., 2021). However, various reviews investigating user priorities and capabilities, and the subsequent design requirements for UL assistive devices attempt to provide generalized guidance (Anderson, 2004; Sarac et al., 2019; Boser et al., 2020; Kobbelgaard et al., 2021; Meyer et al., 2021).

### 3.1 Functional and practical requirements

**Figure 5:**
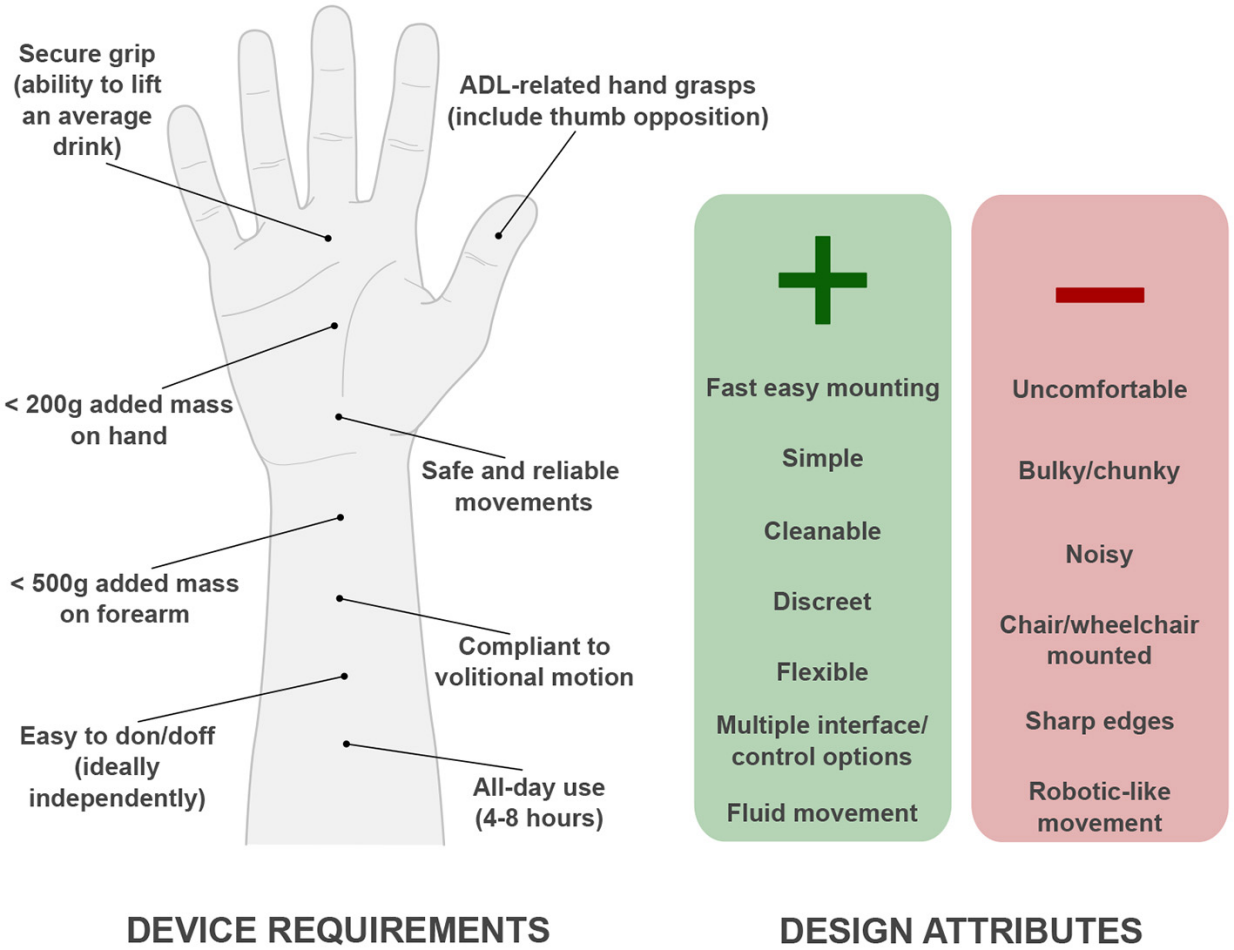
Summary of UL assistive technology user requirements, and corresponding design attributes which users consider as positive or negative (Boser et al., 2020; Kobbelgaard et al., 2021).

A convenient table of functional requirements for an assistive hand exoskeleton is documented by Boser et al. (2020). In summary, the key engineering requirements suggest that a device should: enable a variety of grasp patterns enabling ADL functionality, largely encompassing food and drink preparation and consumption; have a minimum grasp strength capable of lifting an average drink; add a maximum of 200 g additional mass on the hand with potentially up to 500 g on the forearm; and have a maximum bulk size equivalent to that of a ski glove (Boser et al., 2020). Figure 5 illustrates an outline of user requirements.

However, in the authors' experience, clinicians report difficulties of donning a glove-based apparatus on individuals with the effects of a stroke, and particularly for those with apparent spasticity and high tone. In these cases, forced finger extension is often required to fit the device; the action of which can further increase the effects of tone, making subsequent rehabilitation and use of the impaired arm more problematic. Hence, this may also interfere with the effective use of the device after fitting such a glove-based assistive system. Therefore, while the ski glove comparison may give guidance on the acceptable bulk size that may be permissible to users, it should be recognized that a glove may not provide a suitable interface, particularly for users with UL spasticity and high tone.

Additionally, a study of people with chronic UL paresis from a stroke determined that they desired activity-focused goals, working toward independent completion of ADLs, rather than impairment-based interventions, such as exercises for stretching or increased RoM (Waddell et al., 2016). Therefore, an assistive device that can encourage the use of an impaired UL in everyday tasks, may also assist in occupational rehabilitative measures. Accordingly, reports suggest that users may be more permissive of the time spent training and donning/doffing a device which is capable of functional rehabilitative effects (Brown-Triolo et al., 2002). However, sources also cite long training and donning/doffing times as strong risks to cause device abandonment, often due to caregivers prioritizing critical tasks over time spent fitting AT (Kobbelgaard et al., 2021). Hence, ease and simplicity in mounting a device is crucial to ensure practicality and regular usage, but may have been overlooked as a critical design factor. A limitation of user requirement surveys in this field has been that the opinions of caregivers and family members have not generally been included in defining the requirements, yet they are suggested to be instrumental in the lives of the potential users (Kobbelgaard et al., 2021). Therefore, it is important to consider that the functional device requirements are emphasized in reports of user needs, while the importance of the practical aspects of daily living and use may be under-reported and, therefore, undervalued.

Beyond functional and engineering considerations, studies have also explored user perspectives to qualitatively identify the desired attributes of an UL assistive device (Kobbelgaard et al., 2021). As illustrated in Figure 5, many of these preferences center on the convenience and practicality of using AT. Correspondingly, users emphasize the importance of minimizing device visibility and the ease of integration into daily routines; expressing that the functionality and overall size may be preferentially skewed toward performing smaller and potentially repetitive activities, over maximizing lifting capabilities, in order to meet these priorities (Kobbelgaard et al., 2021). While these insights are more challenging to translate into precise design specifications, they provide guidance on which aspects should be prioritized.

#### 3.1.1 Inspiration from lower limb electrical stimulation

Despite clear functional and physiological differences in upper and lower limbs, the success of commercial ES devices for lower limb applications, such foot drop correction systems, may offer valuable design insights for UL technologies. These systems typically target the common peroneal nerve or tibialis anterior muscle, and have proven to be practical, cost-effective, and user-friendly for long-term use (Taylor et al., 2013; Gil-Castillo et al., 2020). Their practicality—often involving a single-site transcutaneous stimulation system embedded in a wearable cuff, with discreet sensor integration retained in footwear—aligns with preferences identified in user-centered studies; where convenience and usability factors, such as aesthetics, mounting ease and location, and minimal visibility, are considered critical for user acceptance (Kobbelgaard et al., 2021). Adopting similar design principles and priorities could, therefore, enhance the usability and adoption of UL assistive solutions.

### 3.2 Sensory aspects

Alongside motor deficits, somatosensory impairments are a common consequence of a stroke and SCI (Connell et al., 2008; Carlsson et al., 2018; Höller et al., 2017). Since transforming sensory input into motor output is integral to volitional human motor behavior (Tresilian, 2012), it corresponds that UL sensory impairment has a highly negative impact on daily life. Yet it often lacks clinical and rehabilitative attention (Carlsson et al., 2018). In addition, proprioception deficits can contribute to difficulties with joint coordination and muscle control (Findlater and Dukelow, 2017), which are important for functional UL use. Additionally, the absence of sensory input can lead to changes in the central nervous system (CNS), including cortical alterations, where such maladaptive neuroplasticity may hinder rehabilitation efforts (Höller et al., 2017)

When ES is used to electrically evoke the sensory volley and therefore target CNS circuits, rather than directly recruiting motor units, it may enhance neuromuscular function for rehabilitation (Bergquist et al., 2011). Emerging evidence suggests that ES techniques may provide rehabilitative effect on proprioception in individuals with chronic stroke (Tashiro et al., 2019). Similarly, research suggests that early application of sensory stimuli involving CNS pathways may also be beneficial in SCI rehabilitation (Höller et al., 2017).

However, the overall evidence is mixed, suggesting there may be nuances in the applications requiring further investigation. A systematic review of studies and RCTs found low- to moderate-quality evidence that, in general, somatosensory stimulation did not improve UL motor function in individuals post-stroke (Grant et al., 2017). Notably though, the reviewed ES protocols varied widely in the stimulation parameters used and treatment dosage. Subsequently, a sensitivity analysis on a subset of the studies revealed that protocols which applied stimulation above the sensory threshold, and were delivered before or during task practice, provided high-quality evidence of improved UL activity in individuals with chronic stroke (Grant et al., 2017). Additionally, a more recent review reported that sensory stimulation applied to peripheral nerves showed positive effects in UL functional recovery following a stroke, whether applied independently or alongside motor training (Fernanda Silva et al., 2024). Although further research is needed to determine whether motor training was the significant contributing factor in the reported improvements, these findings suggest that sensory-targeted stimulation applied nearer the motor threshold, or strategies combining sensory and motor stimulation with functional motor training tasks may offer some degree of rehabilitation.

Correspondingly, a clinical trial investigating ES which was delivered via a neuroprosthetic device to elicit functional movements alongside sensory stimulation, demonstrated improvements in both sensory and motor function in individuals post-stroke, along with more positive personal perceptions of the impaired arm (Crema et al., 2022). This further suggests the potential of integrated sensorimotor ES approaches, and highlights that delivering such an approach via a neuroprosthetic or assistive device may be a beneficial strategy. Nevertheless, optimal stimulation parameters, electrode locations and protocol designs for achieving the most effective outcomes remain inconclusive; which warrants further research. In parallel, increased research attention on incorporating sensory stimulation into assistive neuroprosthetic devices may aid in fulfilling the need for greater sensory-focused interventions, alongside UL motor assistance and training (Carlsson et al., 2018).

## 4 Research challenges in upper limb ES

### 4.1 Overview

Despite ongoing advancements in assistive neuroprostheses, several key challenges remain in the use of ES for the upper limb in particular. A critical challenge is in achieving robust and reproducible performance over extended uses, both within a single session over several hours, and across multiple sessions involving the removal and reapplication of the system (Triolo and Nathan, 1996). This performance should be achieved while also maintaining the characteristics of being a convenient, wearable system, including having an accessible and simple method for fitting the device. Alongside this, several practical aspects that users expect, such as ease of cleaning, maintenance and minimal visibility, raise further constraints.

**Figure 6:**
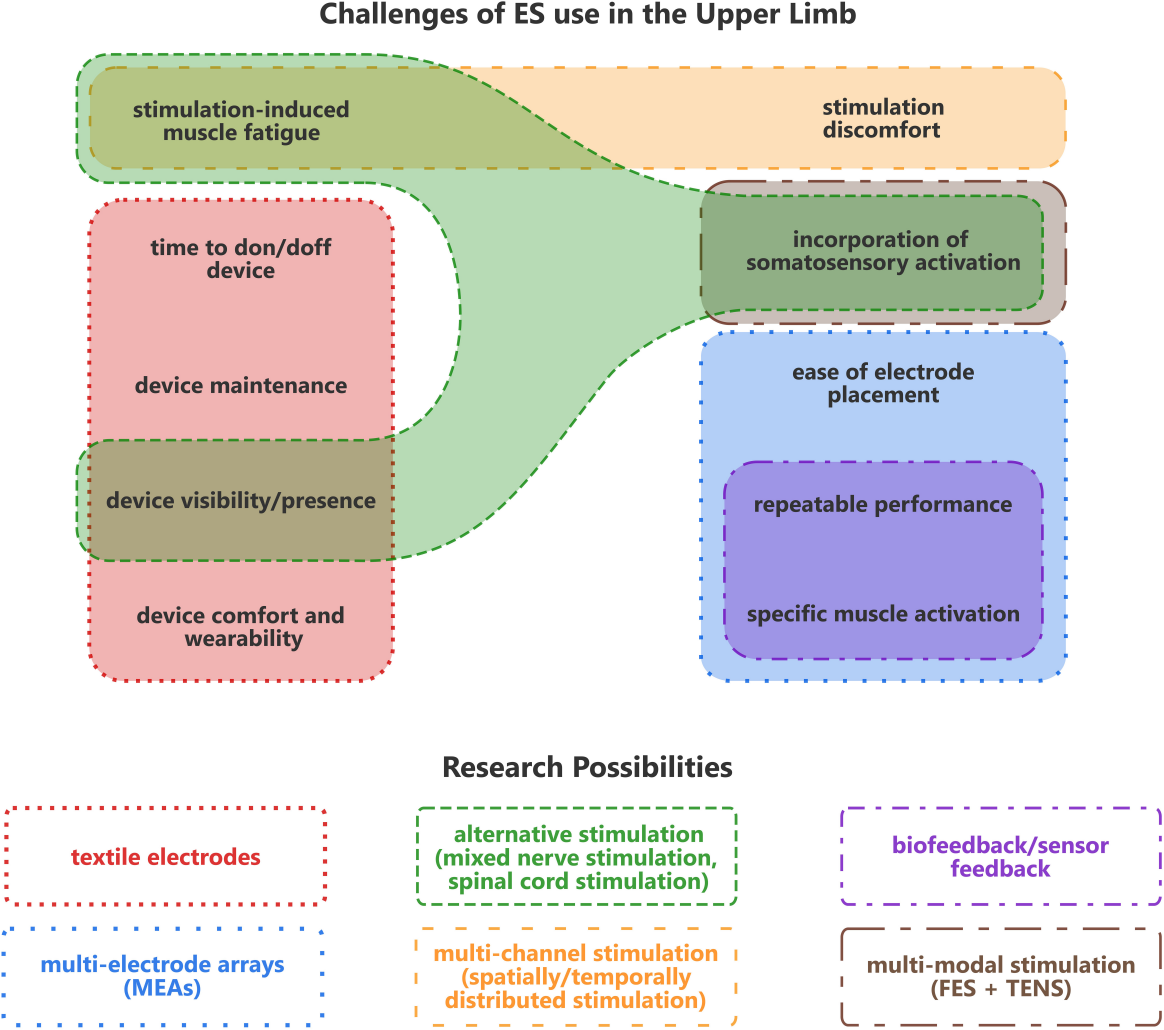
Summary representation of the challenges for using ES on the upper limb, and possible areas of research which aim to address them. Note that this does not detail the full interdependencies and complexities of the challenges and opportunities.

Figure 6 represents a summary of the main challenges associated with the use of ES devices for the UL, and highlights corresponding research areas aimed at mitigating them. Although some research efforts address multiple challenges, as shown, no single approach provides a complete solution in fulfilling all user needs. Consequently, a combined strategy is likely to be necessary. However, given the added complexity of an approach integrating several technologies, it may be necessary to prioritize addressing certain challenges over others to achieve an optimal balance between the device functionality and its practical usability.

Several of the challenges highlighted, such as device comfort, wearability, time to don/doff, and the ease of setup and electrode placement may be classified under a broader category encompassing “device usability.” As discussed in Section 3, these practical usability factors may be undervalued in their importance for enabling the successful adoption and operational use of a hybrid UL assistive device for in-clinic and at-home use. Overall, for upper limb ES to be effective in an assistive capacity, mitigating against stimulation-induced fatigue to enable long-term daily use, as well as improving the device usability aspects are two crucial challenges to address.

### 4.2 Device usability

For a wearable assistive device, it is critical to develop a system not only with the desired functionality, but while also keeping the design convenient and “usable”; being simple to learn, be set-up, worn, used and maintained over its product lifecycle (Meyer et al., 2021). In wearable devices a key region to manage usability factors is in the interface between the user and the device. Within ES applications specifically, this particularly applies to the electrode interface.

The incorporation of long-term wearable electrodes, which may be in the form of textile-based electrodes, is likely to be of key importance in facilitating the usability of ES within neuroprostheses. Textile electrodes can be composed of conductive thread woven into a fabric electrode, or more generally termed textile-based electrodes, consisting of conductive materials, such as carbon-loaded silicone rubbers, set into a wearable fabric (Euler et al., 2022). Such reusable and wearable electrodes could improve the maintainability and ease of set-up of devices over gel and hydrogel-based electrodes that are typically used at present. Textile-based electrodes are being used both as positionable individual electrodes, which can be attached and adjusted on a user-specific garment (Stewart et al., 2019), and as multi-electrode arrays (MEAs) (Yang et al., 2014; Moineau et al., 2019). Each of these approaches aim to improve the method for achieving optimal stimulation locations, while also enhancing wearability and reducing device set-up times.

Research into reusable wearable electrodes is demonstrating promising developments to the field. A comparative study found that applying moisturizing lotion to the skin, prior to the use of silicone rubber electrodes integrated within a textile garment, can achieve similar performance to hydrogel electrodes (Jafari et al., 2025a). This approach may reduce the discomfort often associated with using dry electrodes, and offer a more practical and longer-lasting alternative to existing electrode wetting techniques. However, the study examined larger electrodes on the lower limb quadriceps muscle. Therefore, verification is needed on UL muscles, where discomfort is more prevalent due to typically requiring smaller electrodes and, therefore, higher current densities. Furthermore, the performance was tested for up to 40 min, whereas AT likely demands several hours of use. Consequently, the long-term performance of both wet and dry textile-based electrodes warrants further research, and improvements to support frequent and extended use-times in UL assistive devices (Euler et al., 2022).

For UL applications, the use of MEAs, and particularly in combination with a method of feedback control, offers a promising option for automating appropriate electrode placement. This could help reduce the onus of suitable electrode placement on the user, while enabling specific and repeatable performance. MEAs have also been proposed as a method for improving UL muscle activation selectivity and user adaptability by employing smaller, configurable electrodes that allow for more precise targeting of individual muscles or specific activation patterns (Koutsou et al., 2016). However, this is at the expense of increased system complexity, as MEAs expand the parameter-space, adding further variability and necessitate the use of calibration procedures. To enable practical usability of the device in daily contexts, calibration routines will need to be highly time-efficient, and should have limited active stimulation times so as not to exacerbate ES-induced fatigue prior to use of the device. Achieving an automated set-up for calibrating and guiding electrode placement, will likely require the integration of additional wearable sensors and feedback systems (Usman et al., 2020). However, this adds further complexity to the device.

Overall, a combined use of MEAs, biofeedback, and textile-based or other reusable electrode technologies, may be necessary to address several of the multifaceted challenges of assistive ES for the UL, as illustrated in Figure 6. However, combining each of these components with conventional FES techniques, in a single wearable system, is likely to produce a relatively substantial device. For instance, to activate the extrinsic muscles for enabling hand motion, the stimulation and any feedback system would primarily need to be situated on the forearm and hand. This configuration may be overly cumbersome, conspicuous, and difficult for many users to don and doff—particularly independently. Hence, without integrated and holistic user-centered design considerations, the inclusion of MEAs and biofeedback with FES could ultimately compromise the device usability, and subsequently the level of user acceptance and adoption.

### 4.3 Stimulation-induced fatigue

In addition to usability, addressing stimulation-induced muscle fatigue also remains a crucial challenge. Generally across both upper and lower limb applications, strategies to mitigate fatigue involve; optimizing electrode placement, tuning and optimizing stimulation waveforms and parameters, utilizing MEAs to provide spatially and temporally distributed multi-channel stimulation, and incorporating feedback mechanisms aiming to preferentially recruit fatigue-resistant motor units (Ibitoye et al., 2016).

By providing temporal and spatial variation using an appropriate multi-channel stimulation strategy, MEAs may be employed to counter FES fatigability. This approach aims to mimic a more natural, asynchronous firing pattern and disperse motor unit recruitment to promote more sustainable muscle activation (Crema et al., 2018; Wiest et al., 2019). While methods using MEAs to reduce fatigue through spatial variations in the stimulation pattern have shown promise, some challenges of discomfort and reduced torque remain (Wiest et al., 2019). Though more recently, a study comparing spatially distributed sequential stimulation (SDSS) with single electrode stimulation during isometric muscle contractions, attributed discrepancies in reported torque to the influence of stimulation intensity; whereby SDSS demonstrated superior fatigue resistance and torque output at moderate stimulation intensities (70–85 mA), but with a diminishing advantage as the intensity increased (Jafari et al., 2025b). This suggests that SDSS protocols may be beneficial in use-cases employing lower and moderate stimulation intensities, although its effectiveness during dynamic muscle contractions—as would be needed for assistive applications—warrants further investigation.

These techniques are also more typically studied and applied in lower limb applications, where larger muscles provide a greater surface area over which to provide spatial variations in the stimulation pattern, or to target different motor units within the same muscle (Ibitoye et al., 2016; Wiest et al., 2019). In contrast, upper limb applications present additional challenges; the target muscles are smaller, and several of which lie in deeper fascial compartments, therefore are less accessible to transcutaneous stimulation. Nevertheless, the use of spatially distributed low-frequency stimulation delivered via an MEA has been demonstrated on the extrinsic finger flexor muscles in the forearm. Results showed this approach could approximately double the time to fatigue onset, compared to a more conventional FES protocol (Popović Maneski et al., 2013). However, some practical challenges remain; generally wrist flexion was simultaneously induced, and an initial calibration setup was required to identify the effective electrodes for each desired motion. The calibration involved stimulation applied prior to device-use and additional monitoring sensors to be worn over the fingers. Furthermore, the effectiveness of the selected electrodes could change with variations in forearm rotation, which may necessitate ongoing calibration adjustments to maintain a consistent output. As a result, adaptive or alternative strategies may be required to effectively reduce and manage stimulation fatigue in upper limb ES applications.

## 5 Research opportunities in upper limb ES

Given the complexity and interdependencies of the challenges associated with using ES for the upper limb, there are opportunities to explore alternative approaches that may better align with user needs. The complexity of wearable devices appears to be a strong limiting factor to their adoption (Meyer et al., 2021). Therefore, strategies aimed at reducing overall system complexities, could significantly enhance the usability and subsequent uptake over current designs. Equally, addressing stimulation-induced fatigue is essential for an assistive device with desired use-times potentially up to 8 h (Boser et al., 2020). Although various methods for mitigating fatigue in FES are under active investigation, their translation to UL applications remains somewhat limited due to the anatomical and practical differences in comparison to lower limb muscles.

Previous reviews have primarily focused on evaluating FES techniques for UL rehabilitation and eliciting functional motion (Khan et al., 2023; Sousa et al., 2022; Eraifej et al., 2017), or on neural interfaces and control strategies (Vidaurre et al., 2023; Losanno et al., 2023). However, to the authors' knowledge, reviews assessing different ES strategies that can elicit functional UL motion and, therefore, may be applied within an assistive device are currently lacking.

As several key challenges remain in delivering ES effectively in UL applications and assistive devices, there is opportunity to further explore and refine potential stimulation parameters and protocols. As illustrated in Figure 6, alternative ES techniques may facilitate bridging the currently fragmented areas of research possibilities, and thereby offer a more cohesive and integrated solution to UL devices. Accordingly, the remainder of this article identifies alternative ES paradigms capable of eliciting UL motor responses, and which may offer potential advantages and avenues for future research and innovation in this field.

### 5.1 Overview of alternative ES strategies

Alternative ES strategies extend beyond conventional motor-point FES techniques, with options to instead target neural structures more proximally along the nerve pathway. These approaches can involve the stimulation of mixed sensorimotor nerves, containing both afferent and efferent nerve fibers, or stimulation of the spinal roots. Although such protocols are being explored in research, their application in UL contexts has predominantly focused on the therapeutic benefits and neuromodulatory outcomes, rather than assistive function. Nevertheless, emerging evidence has demonstrated such alternative ES approaches may also be capable of eliciting UL motion, indicating a potential for their use in assistive applications (Shin et al., 2017; Zheng and Hu, 2020a).

To better understand the mechanisms underpinning these strategies, it is essential to examine how these stimulation sites and parameters influence motor recruitment pathways, and expected motor behavior.

#### 5.1.1 Peripheral and central recruitment mechanisms

Electrical stimulation protocols can be tailored to elicit motor responses via central recruitment pathways, thereby evoking muscle contractions with more natural behavior using reflex pathways (Dideriksen J. et al., 2017). These central pathways may be engaged by exploiting particular stimulation sites and waveform parameters to target afferent fibers, thereby inducing motoneuron recruitment trans-synaptically, via the sensory volley (Collins, 2007; Bergquist et al., 2011). Although central reflex pathways are ordinarily associated with generating a transient motor response, electrically stimulated contractions are sustained for the entire stimulation duration, and in the lower limb, have been shown to generate torque output (Collins, 2007). If this effect can be demonstrated to generate consistent functional motor responses, this effect may, therefore, be applicable in assistive applications.

**Figure 7:**
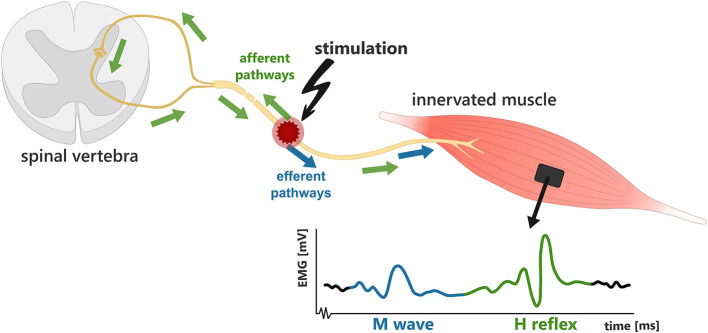
Illustration of the M-wave (blue) and H-reflex (green) pathways following electrical stimulation to a peripheral nerve (Eftekhar et al., 2018). An example electromyography (EMG) trace from the innervated muscle, following stimulation, shows an example where both the M-wave and H-reflex are elicited.

Typically, the incorporation of central reflex pathways in a stimulated motor response, termed the H-reflex, is elicited by stimulating over a muscle using parameters that preferentially activate Ia afferents (Dideriksen J. et al., 2017). With electrodes placed over a muscle, the H-reflex can be selectively targeted using lower stimulation current intensities and higher pulse rates (Dideriksen et al., 2015). Alternatively central reflex pathways may also be activated through proximal stimulation of peripheral sensorimotor nerves (Bergquist et al., 2011). The stimulation of sensorimotor nerves, containing both afferent and efferent neurons, can thereby facilitate motor behavior directly through motor axon stimulation (M-waves), and indirectly via central pathways through the activation of afferent sensory nerves (H-reflex) (Dideriksen et al., 2015; Zheng and Hu, 2019), as illustrated in Figure 7. Muscle contractions can, therefore, be generated by a combination of both M-wave and H-reflex components, or individually, depending on the characteristics of the applied stimulation (Klakowicz et al., 2006). For assistive applications, this combined effect may be beneficial in supporting the functional torque output, while also enabling the potential benefits arising from engaging central pathways.

Existing evidence indicates that appropriate engagement and conditioning of the H-reflex can support rehabilitation, improving function without adverse effects (Eftekhar et al., 2018). As the H-reflex is understood to evoke more synergistic, or biomimetic, muscle activation it may also offer greater fatigue-resistance than conventional FES stimulation strategies (Bergquist et al., 2013; Dideriksen et al., 2015). In lower limb studies involving the soleus muscle and tibial nerve, stimulation patterns incorporating the H-reflex exhibited greater fatigue resistance in participants with an SCI (Bergquist et al., 2013). Comparably, in the upper limb, the use of proximal stimulation of sensorimotor nerves which incorporated the H-reflex, has also demonstrated a slower fatigue rate in stimulated finger flexion compared to motor point FES (Shin et al., 2018). However, this study was conducted on participants without a history of neuromuscular disorders, therefore further research is needed to evaluate the effects of H-reflex conditioning in UL muscles across diverse clinical populations.

Alongside generating a motor response, targeted stimulation to elicit the H-reflex may also provide an inhibitory effect to antagonist muscles, thereby promoting more coordinated movement patterns (Nakagawa et al., 2020). For example, down-conditioning of the H-reflex may aid in reducing flexor over-activation (Eftekhar et al., 2018), which could be beneficial for those with hypertonia. Similarly, proximal nerve stimulation targeting type Ia-afferents has also demonstrated inhibitory effects to antagonistic muscles (Burridge and McLellan, 2000); a mechanism that has also been employed in tremor suppression stimulation strategies (Dideriksen J. L. et al., 2017). These attributes provide further avenues of research investigation, both as an individual therapeutic intervention, or for possible incorporation with assistive ES; particularly as some individuals with UL impairments present with spasticity and hypertonia.

While most research on the H-reflex has focused on the lower limb, there is some work which examines its effect in the flexor carpi radialis (FCR), a wrist flexor muscle (Phadke et al., 2012; Eftekhar et al., 2018). This disparity is particularly significant to address given the functions of the cervical spinal cord affecting UL nerves vary significantly from the neural functions of the lumbar region and lower limbs (Rossi-Durand et al., 1999), which may lead to differences in the neuromuscular response. For instance, the FCR muscle demonstrated a faster recovery profile compared to the soleus muscle in the lower limb (Rossi-Durand et al., 1999). Although the H-reflex has been investigated in some other UL muscles, such as the biceps brachii, extensor carpi radialis and thenar muscles, this has focused on diagnostic applications rather than direct effects on motor output (Burke, 2016).

Considering motor output results, previous studies have demonstrated that the H-reflex can be measured reliably in the FCR muscle, on separate days, and in both healthy and post-stroke participants (Phadke et al., 2012). However, other findings indicate that maintaining a stable posture and controlled environment are essential, as changes in joint angle as well as muscle contractions in other limbs can influence the H-reflex measurement and its physiological effects (Eftekhar et al., 2018). Therefore, further investigation is needed to determine whether functional UL motion may be reliably driven through targeted stimulation of the H-reflex alone.

Instead, methods combining direct (M-wave) and central (H-reflex) recruitment mechanisms by stimulating peripheral sensorimotor nerves proximally, rather than solely targeting type I afferents at the muscle level, may offer a more reliable alternative for generating UL motor responses, while including central involvement. This combined strategy may enable the elicitation of functional motion while simultaneously leveraging some of the potential benefits from including central recruitment pathways to enhance the effectiveness of assistive ES interventions. However, further research is required to fully understand the feasibility and efficacy of this approach in upper limb ES, and to evaluate its applicability across variations in environment and diverse user populations.

#### 5.1.2 Motor point stimulation (MPS) and peripheral nerve stimulation (PNS)

While central recruitment mechanisms can be invoked via stimulation over a muscle motor point using appropriate waveform parameters to target Ia-afferents (Collins, 2007), there is a limit to how large an H-reflex may be generated with increasing stimulation current, before the motor threshold is reached (Burke, 2016). The increase in M-wave then prevents further increases in the H-reflex due to collision blocking from antidromic travel in the motor axons (Burke, 2016). Hence, a target or balance point of central and peripheral recruitment would need to be considered in designing such a stimulation protocol. Therefore, when stimulating over a muscle motor point with the primary aim to elicit motion for assistance, there is likely to be minimal central involvement.

Previous research has sought to characterize the recruitment mechanisms for varying stimulation parameters as well as differences from variations in electrode location. These studies have demonstrated that the H-reflex can be evoked more easily when stimulating over a nerve trunk than over the muscle belly; with maximal H-reflex curves over three times larger from nerve stimulation compared to over a muscle motor point, as tested in lower limb muscles (Bergquist et al., 2011).

**Figure 8:**
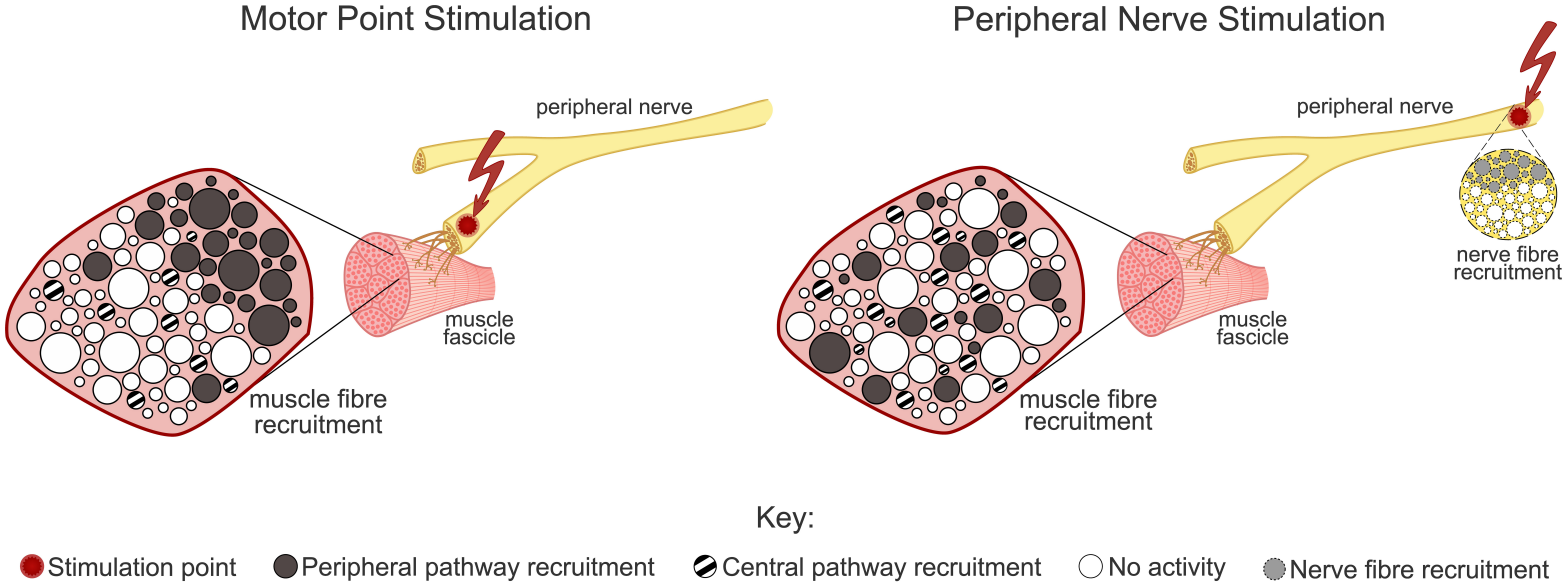
Representation of the proposed effect on central and peripheral pathways for muscle recruitment between motor point stimulation (MPS) and peripheral nerve stimulation (PNS) sites (Bergquist et al., 2011). A more peripheral stimulation location is suggested to provide greater spatial variation in direct muscle fiber recruitment as well as increased recruitment via central pathways.

The comparative effect on the muscle fiber recruitment pattern of motor point stimulation (MPS), which predominantly uses peripheral pathways, vs. peripheral nerve stimulation (PNS), which involves both peripheral and central pathways, is illustrated in Figure 8. As highlighted, the use of PNS may provide more spatial resolution to muscle fiber recruitment, as well as an increase in recruitment via central pathways, such as through the incorporation of the H-reflex. In contrast, when MPS is used to elicit a motor response, studies suggest that it primarily stimulates the motor nerve, and may not evoke a prominent H-reflex response, thereby providing limited central recruitment (Nakagawa et al., 2020).

Conventionally FES is applied using an active electrode over the target muscle belly, ideally over the muscle motor point (Gobbo et al., 2014); therefore may be classified as MPS. This technique produces maximal muscle contraction and torque output with a minimized stimulation current (Gobbo et al., 2014). Hence, likely for these reasons, MPS-style FES has typically been used in UL assistive applications. This assumes a priority for maximizing the motor and torque output. However, from reports of user requirements, this may not fully align with user priorities, for whom other aspects of ES devices, such as the usability and practical aspects may take higher precedence than achieving maximal torque output (Kobbelgaard et al., 2021).

In the upper limb, PNS is more typically applied for its analgesic and neuromodulatory effects in managing chronic pain (Ong Sio et al., 2023), where it is typically delivered via percutaneous or implanted electrodes using low-frequency, tonic stimulation protocols targeting afferent nerves (Stewart et al., 2023). However, transcutaneous ES protocols targeting the stimulation of UL peripheral nerves can be adapted, and subsequently applied above the motor threshold, thereby eliciting joint motion and torque (Shin et al., 2017). Such stimulation protocols, applied over the nerve supply, may also be referred to as functional neuromuscular stimulation (Friederich et al., 2022; Tharu et al., 2023).

Existing studies have demonstrated the possibility of utilizing transcutaneous PNS for evoking UL motor behavior using proximal electrode locations over the supraclavicular fossa and cervical spinal vertebrae (Zheng and Hu, 2020a), and over proximal UL nerves; the median, ulnar (Shin et al., 2017) and radial nerves (Zheng and Hu, 2019). The details of which are discussed in Sections 5.2 and 5.3. Using such proximal stimulation strategies, aiming for increased central involvement in assistive ES protocols may enable the design of a better suited device, with the potential for prioritizing fatigue mitigation, synergistic motion, and a reduced number of stimulation sites.

#### 5.1.3 Synergistic activation

Unlike motor point FES, which aims to activate individual muscles, PNS, when applied proximally, often results in coarse movements that can involve several muscles. This produces less specific muscle activation, instead eliciting motion likely based on muscle synergies at the functional group level (Yan et al., 2025).

Often, increasing muscle selectivity is a primary goal in UL electrical stimulation research. However, the user needs suggest that highly selective activation may not be a direct priority, where synergistic, coordinated motion is likely to be more immediately beneficial. Correspondingly, evidence suggests that the benefits from FES are greatest when UL muscles are used synergistically (Sousa et al., 2022). Therefore, prioritizing synergistic motion over selective muscle activation may be more advantageous for users of UL assistive devices. This may be particularly true while more intuitive control methodologies, which could better enable more selective control of UL postures and movements, such as closed-loop electromyography (EMG) feedback control and brain-computer interfaces (BCI), are largely still in research stages.

Synergistic reaching motions have previously been elicited using conventional FES techniques, citing possible benefits to motor recovery post-stroke (Niu et al., 2019). However, this required numerous electrodes to be placed on muscles over much of the upper limb and chest to achieve a reaching motion, yet still neglected to include hand or wrist functionality. Furthermore, the placement of these FES electrodes prevented the use of EMG due to spatial constraints on each target muscle (Niu et al., 2019). This highlights practical challenges of translating synergistic FES approaches into wearable assistive technologies, and particularly when integrating stimulation with EMG for biofeedback control.

In contrast to conventional FES, proximal peripheral nerve stimulation can activate motoneurons via synaptic transmission in the spinal cord, and is thereby thought to engage the mechanisms underlying those used in voluntary and reflex motions (Bergquist et al., 2011; Dideriksen J. et al., 2017). This approach can facilitate the recruitment of synergistic muscle groups and deeper muscles, which may also reduce fatigue through spatial variation in muscle fiber activation (Shin et al., 2018). These characteristics may be particularly advantageous in UL applications, where conventional FES is often limited to engaging superficial, and typically the extrinsic muscles.

This limitation is especially relevant in the context of hand function, where intrinsic muscles play a key role. These muscles enable finger abduction-adduction motion and can generate significant moments in finger flexion-extension (Lauer et al., 1999). Similarly, the intrinsic thenar muscles are largely responsible for thumb movement, and work in coordination with extrinsic muscles located in the deep fascial compartment of the forearm (Snell, 2012). Given the importance of thumb opposition in functional grasping and pinching (Dollar, 2014; Boser et al., 2020; Smaby et al., 2004), and the contributions of intrinsic hand muscles, the coordinated activation of both intrinsic and extrinsic muscle synergies is considered fundamental to hand functionality (Santello et al., 2013).

Correspondingly, stimulation strategies that leverage muscle synergies including intrinsic and deeper underlying muscles may enhance the performance of ES-based assistive devices. Equally, ES protocols which can engage several muscles from a single stimulation site may aid in reducing the problem dimensionality, by requiring fewer electrodes to achieve coordinated movements, and lower the overall energy demands (Romero-Sánchez et al., 2019).

### 5.2 Proximal nerve stimulation for upper limb assistance

**Figure 9:**
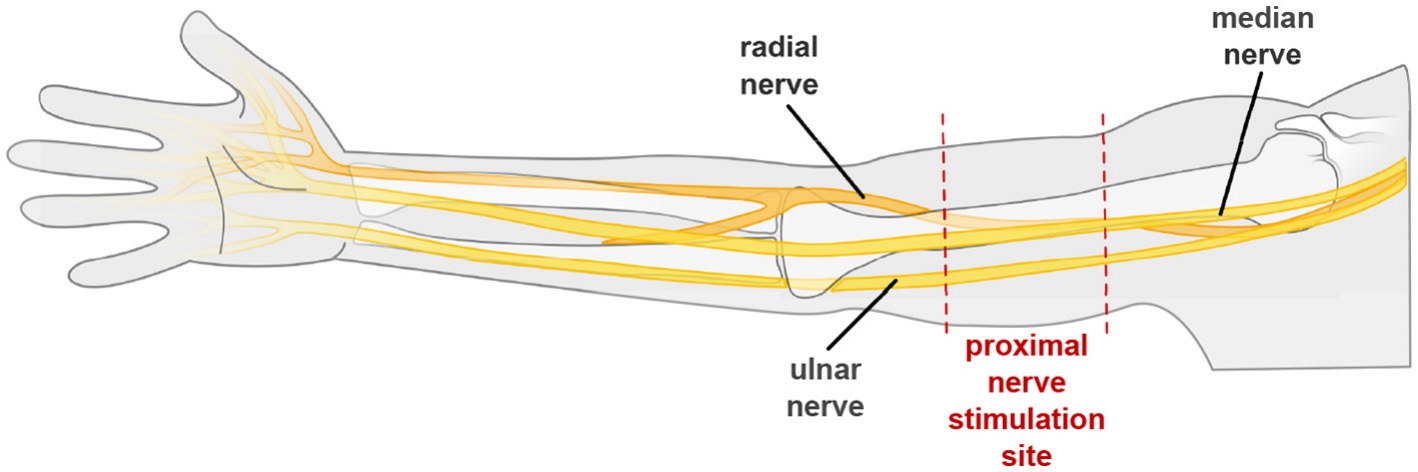
Illustration of the approximate location and pathways of the median, ulnar and radial nerves. The region for applying both cathodes and anodes for proximal nerve stimulation, where the upper limb nerves are relatively superficial, is highlighted within the red dotted lines. The median and ulnar nerve may be targeted on the medial upper arm, with the radial nerve targeted on the lateral side.

Building on these principles, initial research has explored the use of ES at proximal sites on the upper arm to target the median, ulnar and radial nerves for eliciting UL motion (Zheng and Hu, 2019; Shin et al., 2021). These sensorimotor nerves are accessible at relatively superficial locations: the median and ulnar nerves, which primarily innervate the flexor muscles of the hand and forearm (Snell, 2012; Betteridge et al., 2021), can be accessed medially beneath the biceps brachii; while the radial nerve, responsible for primarily innervating the extensor muscles, is accessible posteriorly in the radial groove, approximately midway along the humerus (Zheng and Hu, 2019; Tigra et al., 2020). This region is illustrated in Figure 9. Previous studies using transcutaneous (Shin et al., 2017; Zheng and Hu, 2019) and implanted (Tigra et al., 2020; Azevedo Coste et al., 2022) electrodes have demonstrated that proximal stimulation of these sensorimotor nerves can elicit functional finger flexion and extension, supporting their potential use in UL assistive applications.

#### 5.2.1 Motor and functional responses

Focusing on transcutaneous applications, these studies reported the ability to elicit both independent and coordinated, synergistic UL movements by varying the stimulation location using an MEA positioned in the region shown in Figure 9 (Shin et al., 2017; Zheng and Hu, 2019). The motion was achieved using low stimulation currents; around 4–6 mA, in comparison to approximately 15 mA required for MPS (Shin et al., 2017, 2018; Zheng and Hu, 2019). Proximal stimulation of the median and ulnar nerves was also associated with a delayed onset of fatigue, demonstrating slower decay rates in force and EMG compared to MPS (as indicated by a smaller decay value). Specifically, nerve stimulation showed a decay in force of –12.1 s^−1^ × 10^−3^ vs. –20.4 s^−1^ × 10^−3^ for MPS, and decay in EMG of –1.96 s^−1^ × 10^−3^ vs. –4.33 s^−1^ × 10^−3^, respectively (Shin et al., 2018). Additional details of the stimulation parameters are included in Table 1.

**Table 1:** Comparison table of FES, proximal nerve stimulation and tSCS protocols which demonstrated stimulation-elicited upper limb motion.

**Study**	**Electrode locations**		**Target muscle/s or nerve/s**	**Participants**	**Purpose**	**Stimulation protocol**			
	**Cathode(s)**	**Anode(s)**				**Description**	**Pulse width (**μ**s)**	**RMS current (mA)** §	**RMS current density (mA/cm** ^3^ **)**
**Functional electrical stimulation**									
Crema et al. (2018)	Dorsal hand, thenars, anterior & posterior forearm	Palm, anterior wrist & posterior wrist	Intrinsic hand muscles, extrinsic flexors & extensors	9 (healthy)	Hand function rehabilitation	Assumed rectangular biphasic at 20 Hz^*^	150 (intrinsic) 250 (extrinsic)	0.2 to 3.0	0.1 to 1.56
De Marchis et al. (2016)	All: posterior forearm (alternating rows of active and return electrodes)		Extrinsic hand extensors	8 (healthy)	Assistive hand opening	Biphasic 5 s FES at 30 Hz	500	1.73 (min.)	1.97 (min.)
Niu et al. (2019)	All: Brachium (7 muscles) cathode and anode on muscle belly (approx. 1cm separation)		BB, TBlat, TBlon, AD, PD, PM & BR^†^	6 (ischemic stroke)	Reaching motion rehabilitation	Assumed rectangular biphasic at 20Hz^**^	200	4.24 (max.)	0.27 (max.)
**Proximal nerve stimulation**									
Shin et al. (2021)	All: medial side of brachium (beneath BB† short head)		Median and ulnar nerves	12 (healthy)	Assistive and/or rehabilitative hand function	Biphasic at 25Hz	500	0.47 (min.)	0.60 (min.)
Zheng and Hu (2019)	Brachium (lateral side)	Brachium (medial side)	Radial nerve	8 (healthy)	Assistive and/or rehabilitative hand function	Monophasic 30 Hz burst pulses filled with 10 kHz carrier	80 (burst duration ~1,700)	~0.2 to 2.0	~0.26 to 2.56
**Transcutaneous spinal cord stimulation**									
de Freitas et al. (2021)	Spinal vertebrae (midline over C6, C7 & T1)	(a) anterior neck, (b) clavicles (bilateral), (c) iliac crests (bilateral), (d) mid-back	Lower cervical and upper thoracic spinal roots	10 (healthy)	Testing activation of upper limb motor pools	Monophasic double pulses with 50ms interstimulus interval	2,000	1.96 to 19.6	0.08 to 0.78
Inanici et al. (2018)	Spinal vertebrae (midline C3–C4 & C6–C7)	Iliac crests (bilateral)	Cervical spinal roots	6 (SCI: AIS-B *n* = 2, AIS-C-D *n* = 3 & AIS-D *n* = 1)	Rehabilitation of hand and arm function	Biphasic rectangular at 30 Hz filled with 10 kHz carrier	100 (burst duration 1,000)	14.14 to 21.22	1.41 to 2.16
Zheng and Hu (2020a)	Supraclavicular fossa (right side)	Right of spinal vertebrae (C5–T1)	Lower cervical and upper thoracic spinal roots	6 (healthy)	Assistive upper limb motion	Monophasic pulses at 30 Hz or 120 Hz	600	0.40 to 1.34 (30Hz), 0.80 to 2.68 (120 Hz)	0.20 to 0.67 (30Hz), 0.40 to 1.34 (120 Hz)
Chandrasekaran et al. (2023)	Cervical spinal vertebrae (midline)	Lumbar spinal vertebrae (midline)	Lower cervical and upper thoracic spinal roots	2 (SCI: AIS-A *n* = 1 & AIS-B *n* = 1)	Hand and arm function rehabilitation	Biphasic sinusoidal at 50 Hz	500	70.7 to 159.1	23.6 to 53.0

^§^The root mean square (RMS) stimulation current *i*_*rms*_ and corresponding RMS current density were calculated to enable more valuable direct comparisons between studies with differing waveforms and parameters. RMS current was calculated using following equations for monophasic, biphasic and sinusoidal waveforms, respectively; where *i*_*peak*_ is the reported current, *t*_1_ and *t*_2_ are the negative and positive pulse durations, respectively, and *T* is the total waveform duration. For burst pulse trains utilizing a carrier frequency, a replica of the waveform was generated, and RMS current was calculated using a MATLAB (R2023b) script. $i_{rms}=i_{peak}\sqrt{\frac{t_{1}}{T}}$ (monophasic), $i_{rms}=i_{peak}\sqrt{\frac{\left(t_{1}+t_{2}\right)}{T}}$ (biphasic) and $i_{rms}=i_{peak}\frac{1}{\sqrt{2}}\left(\text{sinusoidal}\right)$.

^†^Muscle abbreviations: *BB*, biceps brachii; *TBlat*, triceps brachii lateral head; *TBlon*, triceps brachii long head; *AD*, anterior deltoid; *PM*, pectoralis major; *BR*, brachioradialis.

^*^Assumed waveform characteristics from related paper (Pedrocchi et al., 2013).

^**^Assumed waveform characteristics from related paper (Qu et al., 2016).

The ability to generate functional, synergistic motion using electrodes covering a more localized stimulation region, and at lower current amplitudes, may offer enhanced wearability and energy efficiency over current stimulation protocols used in hybrid UL assistive devices. However, eliciting extension via radial nerve stimulation proved more challenging in both transcutaneous and implanted approaches (Zheng and Hu, 2019; Tigra et al., 2020), indicating a need for further optimisation of stimulation parameters or electrode location. Despite this, a separate study using implanted electrodes targeting the median and radial nerves proximally above the elbow, successfully demonstrated the ability to elicit both isolated motions and functional hand grasping patterns (Azevedo Coste et al., 2022). Although invasive in nature, this work emphasized the practicalities of using a “single-site” stimulation methodology for generating synergistic motion. Accordingly, a similar approach adapted for non-invasive applications, could improve the practicality and wearability of upper limb AT.

#### 5.2.2 Feedback integration

Stimulating nerves proximally, before they branch into more specialized pathways, may also enable greater activation of sensory fibers involving the dermatomes of the hand and arm (Pan et al., 2023). This may open opportunities for integrating sensory feedback into assistive technologies; an approach which is already under investigation in UL prosthetics (Graczyk et al., 2018; Osborn et al., 2018). As discussed in Section 3.2, incorporating somatosensory feedback with motor assistance may benefit individuals with UL paresis, further supporting the relevance of investigating proximal nerve stimulation in the development of more comprehensive assistive solutions.

Additionally, stimulating over a proximal nerve trunk, such as within the region illustrated in Figure 9, rather than over the target muscle may better support the integration of surface electromyography (sEMG). In conventional motor point FES, both stimulation and sEMG electrodes must be placed on the same muscle (Farina et al., 2004). This can cause difficulties in achieving optimal electrode placement for either system (Niu et al., 2019), as well as subjecting the sEMG recording to significant stimulation artifacts. By contrast, proximal stimulation increases the spatial separation between stimulation and sEMG recording sites. This may potentially reduce the magnitude of, or introduce a temporal delay to, the stimulation artifact that may enable better distinction from the biological sEMG response. This spatial separation may also allow for an increased density of sEMG electrodes over target muscles, enhancing possibilities for monitoring and feedback. The ability to combine stimulation and sEMG effectively, could support the integration of motion intention detection and closed-loop feedback control (Trigili et al., 2019; Dunkelberger et al., 2020), thereby expanding the functional potential of assistive devices.

#### 5.2.3 Further work

As the application of transcutaneous proximal nerve stimulation for eliciting functional UL motion is a relatively immature field of study, there are currently limited reports to enable comparisons and gauge the robustness of the approach. With the early work demonstrating potential, this provides research opportunities to further investigate and verify the principles and initial findings. Factors such as evaluating the protocols in a larger number of participants with diverse characteristics and with upper limb impairments to determine inter-subject variability, alongside assessing the robustness of the approach in providing reliable and consistent assistive UL motion would be beneficial additions to the literature.

Ultimately, both motion and force must be controllable to achieve functional motor assistance. While lower limb studies show a reduced force output from proximal nerve stimulation compared to FES (Dideriksen J. et al., 2017), known physiological differences in upper and lower limb reflex pathways (Milosevic et al., 2019) suggest that a dedicated investigation is needed for UL applications. In particular, characterizing available motions and muscle force generation from proximal nerve stimulation in the UL will be critical in evaluating the suitability of these strategies for assistive technologies.

### 5.3 Transcutaneous spinal cord stimulation (tSCS)

Transcutaneous spinal cord stimulation (tSCS) offers another alternative approach for delivering proximal ES. While cervical spinal cord stimulation has historically involved invasive or percutaneous electrodes targeting upper limb rehabilitation (Waltz et al., 1987), recent studies suggest that cervical-level transcutaneous methods may provide a non-invasive neuromodulation strategy for improving UL voluntary motion, muscle strength and function in people with an SCI (Freyvert et al., 2018; Megía García et al., 2020; Inanici et al., 2021). Moreover, as tSCS appears to act on the functional status of the residual neural networks, its use does not necessarily depend on the etiology of the neuromuscular disorder, hence may also benefit broader clinical populations, such as individuals with cerebral palsy or post-stroke impairments (Barss et al., 2022).

#### 5.3.1 Motor and functional responses

In rehabilitation or neuromodulation, tSCS protocols typically use pulsed frequencies between 1 and 90 Hz, with some studies applying this in combination with a higher carrier frequency, often 2.5–10 kHz (Barss et al., 2022). The cathodic electrodes are generally placed over the cervical or upper thoracic spinal vertebrae (C3–T2), with one or a pair of anodic electrodes typically placed over the anterior neck, back, iliac crests, anterior superior iliac spine (ASIS) or shoulders (de Freitas et al., 2021; Taylor et al., 2021). Depending on the stimulation sites and desired response, current intensities used are typically in the range 30–180 mA (Barss et al., 2022). Reports suggest that the largest elicited motor responses are experienced with cathodes placed over C6–T1, and a single anode over the anterior neck (de Freitas et al., 2021). For further details on rehabilitative tSCS protocols, Barss et al. (2022) and Taylor et al. (2021) provide comprehensive reviews.

While a growing body of literature follows similar tSCS protocols for UL rehabilitation (Freyvert et al., 2018; Inanici et al., 2021; Fleming et al., 2022), one study reports an anodic tSCS protocol specifically aimed at eliciting UL motion. This disparate approach used significantly lower stimulation currents, averaging approximately 6 mA, with cathodes placed over the supraclavicular fossa and anodes over the C5–T1 vertebrae (Zheng and Hu, 2020a); further details in Section 5.4. This configuration evoked EMG responses in distal UL muscles (Zheng and Hu, 2020b), and subsequently, elicited independent and synergistic motions of the fingers, hand, and wrist (Zheng and Hu, 2020a).

The lower current intensities for eliciting motion reported in this protocol, as compared to levels of 20–40 mA for conventional FES, or even higher for typical cathodic tSCS, offer potential advantages in providing more comfortable and power efficient stimulation for assistive, neuroprosthetic applications (Zheng and Hu, 2020a). Additionally, the more proximal location of the electrodes was reportedly unaffected by muscle contractions and UL motion, suggesting it provides a more stable stimulation site during use (Zheng and Hu, 2020a). However, due to the reversal from the typical tSCS electrode polarity, and significantly lower current intensities, it has been hypothesized that this protocol may be primarily stimulating the brachial plexus, rather than the dorsal or ventral roots in the cervical spinal cord (Routledge et al., 2024). This hypothesis corresponds with the findings of a previous study with a similar stimulation configuration, which suggested that excitation occurred distally to the spinal root segments (Schmid et al., 1990). Despite diverging from standard tSCS methods and the current uncertainty surrounding its mechanism of action, this stimulation approach may offer practical advantages for eliciting UL motion. However, these findings are based on a small sample of just six healthy participants. As such, further investigation into this protocol would be beneficial to clarify the underlying mechanisms and assess its potential for assistive applications.

Generally, research investigating the use of tSCS for upper limb motion assistance applications is in relatively early stages, particularly as most current literature focuses on investigating its rehabilitative potential. Therefore, motor response outcomes are generally presented with this emphasis. While the tSCS studies highlighted in Table 1 reported results of motor output, this was assessed using sEMG. Although this indicates potential for eliciting motion, sEMG results alone offer limited insight for evaluating functional motor output, such as force and torque generation, which are key to assistive applications. This suggests a research opportunity to characterize tSCS protocols not only in terms of muscle activation, but also functional motor output. Initial work in this area has used sEMG in conjunction with a robotic exoskeleton to measure levels of both muscle activation and torque generation in response to single pulse tSCS (Mahan et al., 2024). While these results demonstrate that tSCS can elicit UL joint torque, the study was performed in individuals without any form of paresis and reported wide inter-subject variability. This underscores the need for further characterization work and investigation into assessing the functional outcomes of different tSCS protocols alongside muscle activation for UL assistance.

#### 5.3.2 Sensory integration

The effects of tSCS on UL sensation are often under-reported. However, a recent study documented improvements in tactile sensation, assessed by Graded Redefined Assessment of Strength, Sensation and Prehension (GRASSP) tests, in two individuals with a cervical-level SCI (Chandrasekaran et al., 2023). Although preliminary and with limited participants, these findings suggest a potential of tSCS in addressing somatosensory deficits, which are typically overlooked in UL rehabilitation, yet often concomitant with motor impairments (Tashiro et al., 2019).

In assistive applications, integrating methods for involving UL sensation could provide important sensory feedback for aiding grasping. However, concerns remain regarding the effects of collision blocking from antidromic transmission of stimulated peripheral pathways in proximal nerve fibers. This may instead lead to proprioception loss during active stimulation, with a reportedly greater blocking effect the closer the electrode to the spinal cord (Duffell and Donaldson, 2020). While methods of high-frequency low-amplitude stimulation have been proposed to mitigate this effect (Formento et al., 2018), the impact of tSCS on proprioception is seldom addressed in the literature. This suggests opportunities for targeted tSCS research, focusing on assessing motion assistance alongside any sensory and proprioceptive effects.

#### 5.3.3 Trunk stability

A further potential benefit of tSCS in assistive contexts is a possibility to incorporate trunk stability support (Tharu et al., 2023). Evidence suggests that integrating postural control of the trunk with ES-based UL assistance may be essential to better enable larger functional movements, such as reaching (Sousa et al., 2022). As trunk and shoulder stability can often be a limiting factor in achieving functional motion in post-stroke and SCI populations, incorporating trunk activation with tSCS could enhance the assistive functionality of the overall ES strategy.

Previously, upper limb FES combined with trunk support has been used to assist functional task-specific training, which suggested benefits in an integrated approach to UL assistance (Tefertiller et al., 2022). While existing studies have examined the use of tSCS and neuromodulation methods for rehabilitating trunk stability (Rath et al., 2018; Tharu et al., 2023), investigating their integration for providing postural control alongside UL assistance may provide an interesting opportunity for future exploration.

### 5.4 ES parameter comparison

Section 5.1 discussed the potential of ES techniques beyond conventional motor point FES for eliciting UL assistive movements. Existing auxiliary research into proximal nerve stimulation paradigms has shown promise, both in eliciting significant motion, and in the potential to better address some of the user requirements for an UL assistive device. However, there is considerable variation in the stimulation paradigms employed, and many of the existing studies do not directly evaluate functional motor outcomes relevant to UL assistance. To facilitate comparison across ES approaches, Table 1 summarizes the stimulation parameters from several studies including: conventional FES, FES applied via a multi-electrode array, the stimulation of proximal UL nerves, and transcutaneous spinal cord stimulation (tSCS). These studies were selected based on their reported ability to elicit UL motion, indicating their potential for consideration in assistive applications.

Preliminary comparisons suggest that proximal nerve stimulation techniques may be able to elicit UL motion with comparable, or slightly reduced stimulation current intensity relative to FES. A reduction in stimulation current would be beneficial as it could provide a power saving for battery-powered, wearable devices. Though with a limited number of studies available, and the disparity between stimulation paradigms, these comparisons are not conclusive. Additionally, among the tSCS studies, while carrier frequencies with modulated waveforms are typically used, there are suggestions that when current waveforms are normalized for intensity, the carrier frequency has no significant effect (Manson et al., 2020). While many of the tSCS studies quote similar stimulation currents used, there is a range of varying current densities due to the disparity in electrode sizes used. Therefore, further research to better compare and optimize the associated stimulation parameters, such as electrode size and the overall delivered charge and corresponding energy, would be beneficial to assess the applicability of these paradigms.

As highlighted in Table 1, a diverse range of stimulation paradigms have exhibited UL motor responses. Although much of the existing literature focuses on rehabilitative and neuromodulatory outcomes, several protocols have demonstrated the ability to generate movement, presenting an opportunity to explore these ES approaches for integration into assistive technology. Importantly, however, considerable variation in the reported stimulation parameters and relatively low participant numbers necessitates further characterization work to investigate optimal parameter values and verify the outcomes. Additionally, more targeted research is required to evaluate the capacity of these protocols to generate appropriate functional motor and force outputs suitable for UL assistance.

In summary, transcutaneous ES techniques originally developed for rehabilitative applications, such as neuromodulation, may offer distinct advantages when adapted and used in combination within a hybrid assistive device. With a more proximal electrode placement, these strategies may improve the stability of elicited movements with respect to UL motion, reduce the number of required electrodes, and better facilitate integration with biofeedback systems such as EMG. Furthermore, the concurrent advancement of textile-based electrodes offers promising avenues for embedding ES technology under or within clothing, potentially improving user comfort and simplifying donning and doffing procedures—which are identified as important aspects for consistent use of AT.

## 6 Discussion

Published reports of user requirements for UL assistive devices have provided engineering specifications in terms of typical grasp patterns and force outputs required for ADLs, as well as generalized design wishes from potential users. However, most of the literature underplays the importance and significance of carers or support workers who may also be responsible for fitting, adjusting, and removing assistive devices. Practical aspects, such as the ease of electrode placement/positioning, requirements for skin preparation and device cleaning, and ease of fitting the aid as a whole, all play a key role in device uptake and regular usage. Addressing the practicalities of device-use may be key to enabling user uptake and minimizing device abandonment.

Given the marked heterogeneity of the target users for UL assistive devices, a “one-size-fits-all” approach may be impractical. Reports of assistive and rehabilitative devices often lack details on whether a specific condition is targeted, for example paresis or spasticity, which will lead to differing specifications. A generalized AT device would need to adapt to differing and potentially coexisting deficits across users. Accordingly, the combination of different AT modalities, within a single platform, could provide the flexibility required by enabling passive support and active assistance. Passive orthoses could be combined with active mechanical assistance and ES within a hybrid system that provides the flexibility to support a wide range of user needs. However, due to the complexity of combining these approaches in one device, such hybrid systems may have a deleterious effect on usability and practicality if they are not designed with these requirements in mind from the outset. This is more likely to be achievable with a predominantly ES-driven device due to the ability to automatically vary stimulation parameters (e.g., via multi-electrode arrays) to adapt or tune stimulation to account for daily variations in electrode placement or physical function. However, while developments in MEAs and textile-based electrodes demonstrate effective use in research environments, a limited translation beyond this highlights that the practicalities of their use may still need addressing. While MEAs can bring about better selectivity and adaptation between users, this comes at the cost of an increased “problem-space,” with many additional variables to consider and calibrate for.

Instead of conventional FES techniques, the use of stimulation more proximally over upper limb nerves has been shown to elicit synergistic motion, invoking motion from muscle groups, while stimulating from a single area on the UL. Such synergistic motion is likely to elicit coarser, rather than selective hand and arm movements. However, reported discussions with potential users and their carers suggest that practicality may be the greater limiting factor to uptake of AT devices. Hence prioritizing the day-to-day practicalities, such as ease of donning and doffing, could be favored over selective UL control. Reducing the number and coverage needed for stimulation electrode sites, while still enabling collaborative functional motion may be beneficial in reducing the problem-space of upper limb transcutaneous ES. With conventional FES, this would typically involve having to place electrodes over several stimulation sites, or the use of large MEAs, covering much of the UL. Therefore, restricting the active stimulation sites to a smaller or single area may aid the practicality and usability of ES in a neuroprosthetic device.

However, a clear challenge for proximal upper limb stimulation strategies is in eliciting predictable and repeatable motion for a given stimulation setup. The impact of limb position and orientation on FES is well documented, however this is not the case for stimulation over a proximal nerve or for neuromodulatory stimulation protocols typically targeting therapeutic effects. To evaluate use of these protocols for assistive purposes will require additional and specific characterization work, which is currently lacking from the literature. Alongside this, sensory impairment is a common residual of many neuromuscular disorders, yet it is often overlooked in the design of UL assistive devices. In the context of UL prostheses, for example, sensory feedback is considered to be a critical factor in device acceptance and retention (Jabban et al., 2022). Protocols targeting the primary stimulation of afferents, or a mixed approach stimulating both afferent and efferent fibers, show potential for eliciting synergistic motion via central pathway recruitment, and may also have greater fatigue resistance than FES (Shin et al., 2018). Alongside this, incorporating afferent stimulation involving the central nervous system, may be beneficial for neuroplastic recovery (Höller et al., 2017) and provide potential for sensorimotor rehabilitation in relevant clinical populations (Crema et al., 2022; Chandrasekaran et al., 2023). However, further investigation is needed.

In terms of when such a hybrid AT device may be most useful to users, discussions with stroke clinicians suggest that the target time-period is in assisting the transition from the stabilized acute phase of recovery into the chronic stages. It is envisaged that clinicians could use a hybrid device with their clients during in-patient rehabilitation, with follow-up usage in the home environment, thereby easing the transition to out-patient therapy while initially building the user's initial confidence in the device in clinic. This is in agreement with evidence suggesting that, particularly for individuals who have had a stroke, rehabilitative treatment of the affected arm early after the stroke occurrence appears to be most beneficial. Though there is also evidence to suggest that improvements from AT-use may also be seen into the chronic stages—over 6-months post stroke (Farmer et al., 2014). Additionally, research suggests a similarly timed application of AT for people with an SCI may also be beneficial (Readioff et al., 2021). The use of an assistive device which engages synergistic functional motions over more specific joint activation would likely be more apt for applying at this stage of intervention.

In summary, FES has seen extensive clinical use for rehabilitation, and there is evidence of its application in UL assistive technology within research settings. More general ES and neuromodulation techniques, such as proximal PNS or tSCS, have been the focus of intensive research for therapeutic purposes, but have yet to be fully exploited in the clinic or adapted for AT. This paper seeks to demonstrate that within the context of UL assistance it may be appropriate to research and apply a wider range of electrical stimulation protocols not limited to traditional FES, and that this, in combination with passive or active mechanical assistance may improve overall device designs and user outcomes.

## 7 Future research

Future research should prioritize determining optimal stimulation parameters, and characterizing proximal ES strategies for upper limb assistance, with particular emphasis on assessing their capacity to elicit reproducible, functional assistive motions across diverse user populations. Studies should aim to evaluate these approaches in the context of end-user priorities, focusing on the ultimate practical aspects of an assistive device design, such as ease of setup, use and fitting, alongside the functional capabilities of the device. Additionally, examining the potential of proximal ES strategies to support sensory feedback or rehabilitation alongside motor assistance would provide clearer guidance for assistive neuroprosthesis design. If shown to be effective, the integration of sensory or proprioceptive feedback may offer improved solutions to assistive devices.

In broader contexts, given the potential of MEAs to modulate the active electrode location and, therefore, vary the movement type elicited from proximal ES, furthering the development of MEAs using reusable textile-based electrode technology may offer more discreet and convenient solutions for delivering assistive stimulation protocols. In parallel, developing minimally obtrusive wearable or textile-based sensors for recording biofeedback, such as EMG or joint positions, could support the integration of control and calibration strategies. This may be particularly valuable for facilitating the implementation of MEAs in upper limb ES systems, where adaptive methods for calibrating and selecting suitable active electrodes are likely to be necessary to accommodate variations which may arise from the effects of varying limb position, removal and reapplication of the device and inter-subject variability.

## 8 Conclusion

Expected increases in aging and non-communicable diseases globally are likely to increase the need for solutions to the effects of motor dysfunction. In particular, motor impairments in the upper limb have a considerable impact on a person's capacity for independence. Ongoing research into technological solutions to address this includes assistive devices incorporating robotics, orthoses, and electrical stimulation, as well as the combination of these techniques in neuroprosthetic and hybrid devices. The use of ES within UL assistive applications holds promise for enhancing function in individuals with motor impairments, and in aiding lighter-weight hybrid device designs than a sole reliance on robotic systems. Currently, most ES-based assistive devices employ conventional FES techniques. Despite notable advancements in device design, electrode technologies, and control strategies, several key challenges remain that limit the widespread adoption and practical usability of stimulation in UL applications.

This paper has reviewed the use of electrical stimulation for upper limb assistance, highlighting the challenges of conventional FES in managing the specific anatomical demands of the UL, and assessing the user requirements for an UL assistive device. In particular, the analysis of user priorities suggests that challenges with stimulation-induced fatigue and difficulties in practical usability may limit the effectiveness and user adoption of such devices. While research efforts, such as MEAs, biofeedback mechanisms and textile-based electrodes, aim to address these issues, they are typically designed around conventional motor-point FES techniques, which may not be optimal for use in UL applications. Consequently, this review has highlighted alternative forms of ES that have demonstrated the capacity to elicit UL motion, and outlined background information demonstrating their potential use in assistive applications.

Alternative ES paradigms, such as proximally-applied nerve stimulation, spinal cord stimulation, or neuromodulatory techniques, may offer promising options for overcoming some of the limitations experienced when employing FES techniques in UL assistance. Such alternative approaches may offer synergistic muscle activation for improved fatigue management and a more localized stimulation site, which could enhance the practicalities of an overall device design. However, many of these existing protocols are currently in the early stages of development with relation to being applied in an assistive capacity. Initial studies suggest they may offer benefits when applied to UL assistance, but further characterization work is needed to verify the range and reproducibility of functional motions that can be elicited, and to assess their applicability in relevant user populations. Evaluating the feasibility of these ES strategies as potential alternatives to conventional FES, and exploring their integration with existing concepts in UL assistive systems, provides novel research opportunities which may ultimately offer more effective device designs. By aligning the electrical stimulation strategies used in upper limb assistive applications with users' priorities and practical needs, alongside integration into pragmatic designs, future research may offer more user-focused, and impactful neuroprosthetic assistive technologies.

## References

[B1] Ambrosini E. Gasperini G. Zajc J. Immick N. Augsten A. Rossini M. . (2021). A robotic system with EMG-triggered functional eletrical stimulation for restoring arm functions in stroke survivors. Neurorehabil. Neural Repair 35, 334–345. doi: 10.1177/1545968321997769 33655789

[B2] Anderson K. D. (2004). Targeting recovery: priorities of the spinal cord-injured population. J. Neurotrauma 21, 1371–1383. doi: 10.1089/neu.2004.21.1371 15672628

[B3] Anwer S. Waris A. Gilani S. O. Iqbal J. Shaikh N. Pujari A. N. . (2022). Rehabilitation of upper limb motor impairment in stroke: a narrative review on the prevalence, risk factors, and economic statistics of stroke and state of the art therapies. Healthcare 10, 1–20. doi: 10.3390/healthcare10020190 35206805 PMC8872602

[B4] Axelgaard (2025). NMES Electrode Placement. Available online at: https://www.axelgaard.com/Education (Accessed January 20, 2025).

[B5] Azevedo Coste C. William L. Fonseca L. Hiairrassary A. Andreu D. Geffrier A. . (2022). Activating effective functional hand movements in individuals with complete tetraplegia through neural stimulation. Sci. Rep. 12, 1–17. doi: 10.1038/s41598-022-19906-x 36202865 PMC9537317

[B6] Baker L. L. Wederich C. L. McNeal D. R. Newsam C. Waters R. L. (2000). Neuro Muscular Electrical Stimulation: A Practical Guide. Downey, CA: Los Amigos Research & Education Institute, Inc.

[B7] Barss T. S. Parhizi B. Porter J. Mushahwar V. K. (2022). Neural substrates of transcutaneous spinal cord stimulation: neuromodulation across multiple segments of the spinal cord. J. Clin. Med. 11:639. doi: 10.3390/jcm11030639 35160091 PMC8836636

[B8] Bergquist A. J. Clair J. M. Lagerquist O. Mang C. S. Okuma Y. Collins D. F. . (2011). Neuromuscular electrical stimulation: implications of the electrically evoked sensory volley. Eur. J. Appl. Physiol. 111, 2409–2426. doi: 10.1007/s00421-011-2087-9 21805156

[B9] Bergquist A. J. Wiest M. J. Okuma Y. Collins D. F. (2013). H-reflexes reduce fatigue of evoked contractions after spinal cord injury. Muscle Nerve 50, 224–234. doi: 10.1002/mus.24144 24638882

[B10] Bersch I. Fridén J. (2021). Electrical stimulation alters muscle morphological properties in denervated upper limb muscles. eBioMedicine 74:103737. doi: 10.1016/j.ebiom.2021.103737 34896792 PMC8671101

[B11] Betteridge N. Taylor A. Hartley R. (2021). Clinical anatomy of the nerve supply to the upper limb. BJA Educ. 21, 462–471. doi: 10.1016/j.bjae.2021.07.007 34840818 PMC8606604

[B12] Boser Q. A. Dawson M. R. Schofield J. S. Dziwenko G. Y. Hebert J. S. (2020). Defining the design requirements for an assistive powered hand exoskeleton: A pilot explorative interview study and case series. Prosthet. Orthot. Int. 45, 161–169. doi: 10.1177/0309364620963943 33118453 PMC8404210

[B13] Brown-Triolo D. L. Roach M. J. Nelson K. Triolo R. J. (2002). Consumer perspectives on mobility: Implications for neuroprosthesis design. J. Rehabil. Res. Dev. 39, 659–669. 17943668

[B14] Burke D. (2016). Clinical uses of H reflexes of upper and lower limb muscles. Clinical Neurophysiol. Pract. 1, 9–17. doi: 10.1016/j.cnp.2016.02.003 30214954 PMC6123946

[B15] Burridge J. H. McLellan D. L. (2000). Relation between abnormal patterns of muscle activation and response to common peroneal nerve stimulation in hemiplegia. J. Neurol. Neurosurg. Psychiatry 69, 353–361. doi: 10.1136/jnnp.69.3.353 10945810 PMC1737092

[B16] Bussel B. (2015). History of electrical stimulation in rehabilitation medicine. Ann. Phys. Rehabil. Med. 58, 198–200. doi: 10.1016/j.rehab.2015.04.008 26189790

[B17] Camona C. Wilkins K. B. Drogos J. Sullivan J. E. Dewald J. P. Yao J. . (2018). Improving hand function of severely impaired chronic hemiparetic stroke individuals using task-specific training with the rein-hand system: a case series. Front. Neurol. 9:923. doi: 10.3389/fneur.2018.00923 30464754 PMC6234834

[B18] Carlsson H. Gard G. Brogårdh C. (2018). Upper-limb sensory impairments after stroke: self-reported experiences of daily life and rehabilitation. J. Rehabil. Med. 50, 45–51. doi: 10.2340/16501977-2282 29068038

[B19] Chandrasekaran S. Bhagat N. A. Ramdeo R. Ebrahimi S. Sharma P. D. Griffin D. G. . (2023). Targeted transcutaneous spinal cord stimulation promotes persistent recovery of upper limb strength and tactile sensation in spinal cord injury: a pilot study. Front. Neurosci. 17:1210328. doi: 10.3389/fnins.2023.1210328 37483349 PMC10360050

[B20] Collins D. F. (2007). Central contributions to contractions evoked by tetanic neuromuscular electrical stimulation. Exerc. Sport Sci. Rev. 35, 102–109. doi: 10.1097/jes.0b013e3180a0321b 17620928

[B21] Connell L. A. Lincoln N. B. Radford K. A. (2008). Somatosensory impairment after stroke: frequency of different deficits and their recovery. Clin. Rehabil. 22, 758–767. doi: 10.1177/0269215508090674 18678576

[B22] Crema A. Bassolino M. Guanziroli E. Colombo M. Blanke O. Serino A. . (2022). Neuromuscular electrical stimulation restores upper limb sensory-motor functions and body representations in chronic stroke survivors. Med. 3, 58–74. doi: 10.1016/j.medj.2021.12.001 35590144

[B23] Crema A. Malešević N. Furfaro I. Raschellà F. Pedrocchi A. Micera S. (2018). A wearable multi-site system for NMES-based hand function restoration. IEEE Trans. Neural Syst. Rehabil. Eng. 26, 428–440. doi: 10.1109/TNSRE.2017.2703151 28500007

[B24] Crook S. Vanhoestenberghe A. (2024). “Medical device safety and regulatory compliance – a guide for beginners,” in Techniques and Technologies in Electrical Stimulation for Neuromuscular Rehabilitation, Chapter 3, eds. I. Swain, J. Burridge, and T. Street (The Institution of Engineering and Technology), 51–67. doi: 10.1049/PBHE062E_ch3

[B25] de Freitas R. M. Sasaki A. Sayenko D. G. Masugi Y. Nomura T. Nakazawa K. . (2021). Selectivity and excitability of upper-limb muscle activation during cervical transcutaneous spinal cord stimulation in humans. J. Appl. Physiol. 131, 746–759. doi: 10.1152/japplphysiol.00132.2021 34138648

[B26] De Marchis C. Monteiro T. S. Simon-Martinez C. Conforto S. Gharabaghi A. (2016). Multi-contact functional electrical stimulation for hand opening: electrophysiologically driven identification of the optimal stimulation site. J. Neuroeng. Rehabil. 13, 1–9. doi: 10.1186/s12984-016-0129-6 26955873 PMC4782521

[B27] Dideriksen J. Leerskov K. Czyzewska M. Rasmussen R. (2017). Relation between the frequency of short-pulse electrical stimulation of afferent nerve fibers and evoked muscle force. IEEE Trans. Biomed. Eng. 64, 2737–2745. doi: 10.1109/TBME.2017.2671853 28237919

[B28] Dideriksen J. L. Laine C. M. Dosen S. Muceli S. Rocon E. Pons J. L. . (2017). Electrical stimulation of afferent pathways for the suppression of pathological tremor. Front. Neurosci. 11, 1–11. doi: 10.3389/fnins.2017.00178 28420958 PMC5378793

[B29] Dideriksen J. L. Muceli S. Dosen S. Laine C. M. Farina D. (2015). Physiological recruitment of motor units by high-frequency electrical stimulation of afferent pathways. J. Appl. Physiol. 118, 365–376. doi: 10.1152/japplphysiol.00327.2014 25477350

[B30] Dollar A. M. (2014). “Classifying human hand use and the activities of daily living,” in The Human Hand as an Inspiration for Robot Hand Development, Vol. 95, Chapter 10, eds. R. Balasubramanian, and V. J. Santos (Cham: Springer International Publishing), 201–216. doi: 10.1007/978-3-319-03017-3_10

[B31] Doucet B. M. Lam A. Griffin L. (2012). Neuromuscular electrical stimulation for skeletal muscle function. Yale J. Biol. Med. 85, 201–215. doi: 10.1016/j.jelekin.2011.12.005 22737049 PMC3375668

[B32] Duffell L. D. Donaldson N. N. (2020). A comparison of FES and SCS for neuroplastic recovery after SCI: historical perspectives and future directions. Front. Neurol. 11:607. doi: 10.3389/fneur.2020.00607 32714270 PMC7344227

[B33] Dunkelberger N. Schearer E. M. O'Malley M. K. (2020). A review of methods for achieving upper limb movement following spinal cord injury through hybrid muscle stimulation and robotic assistance. Exp. Neurol. 328:113274. doi: 10.1016/j.expneurol.2020.113274 32145251

[B34] Eftekhar A. Norton J. J. McDonough C. M. Wolpaw J. R. (2018). Retraining reflexes: clinical translation of spinal reflex operant conditioning. Neurotherapeutics 15, 669–683. doi: 10.1007/s13311-018-0643-2 29987761 PMC6095771

[B35] Eraifej J. Clark W. France B. Desando S. Moore D. (2017). Effectiveness of upper limb functional electrical stimulation after stroke for the improvement of activities of daily living and motor function: a systematic review and meta-analysis. Syst. Rev. 6, 1–21. doi: 10.1186/s13643-017-0435-5 28245858 PMC5331643

[B36] Euler L. Guo L. Persson N. K. (2022). A review of textile-based electrodes developed for electrostimulation. Text. Res. J. 92, 1300–1320. doi: 10.1177/00405175211051949

[B37] Farina D. Merletti R. Enoka R. M. (2004). The extraction of neural strategies from the surface EMG. J. Appl. Physiol. 96, 1486–1495. doi: 10.1152/japplphysiol.01070.2003 15016793

[B38] Farmer S. E. Durairaj V. Swain I. Pandyan A. D. (2014). Assistive technologies: can they contribute to rehabilitation of the upper limb after stroke? Arch. Phys. Med. Rehabil. 95, 968–985. doi: 10.1016/j.apmr.2013.12.020 24429002

[B39] Feigin V. L. Brainin M. Norrving B. Martins S. Pandian J. Lindsay P. . (2025). World Stroke Organization (WSO): global stroke fact sheet 2025. Int. J. Stroke 20, 132–144. doi: 10.1177/17474930241308142 39635884 PMC11786524

[B40] Feigin V. L. Vos T. Nichols E. Owolabi M. O. Carroll W. M. Dichgans M. . (2020). The global burden of neurological disorders: translating evidence into policy. Lancet Neurol. 19, 255–265. doi: 10.1016/S1474-4422(19)30411-9 31813850 PMC9945815

[B41] Fernanda Silva G. Campos L. F. de Aquino Miranda J. M. Guirro Zuliani F. de Souza Fonseca B. H. de Araújo A. E. T. . (2024). Repetitive peripheral sensory stimulation for motor recovery after stroke: a scoping review. Top. Stroke Rehabil. 31, 723–737. doi: 10.1080/10749357.2024.2322890 38452790

[B42] Findlater S. E. Dukelow S. P. (2017). Upper extremity proprioception after stroke: bridging the gap between neuroscience and rehabilitation. J. Mot. Behav. 49, 27–34. doi: 10.1080/00222895.2016.1219303 27726645

[B43] Fleming N. Taylor C. Etzelmueller M. Gill C. Keeffe C. O. Mahony N. . (2022). “Selectivity of upper limb posterior root muscle reflexes via cervicothoracic spinal cord stimulation,” in 44th Annual International Conference of the IEEE Engineering in Medicine & *Biology Society (EMBC)* (Glasgow: IEEE), 3126–3129. doi: 10.1109/EMBC48229.2022.9871841 36085735

[B44] Formento E. Minassian K. Wagner F. Mignardot J. B. Le Goff-Mignardot C. G. Rowald A. . (2018). Electrical spinal cord stimulation must preserve proprioception to enable locomotion in humans with spinal cord injury. Nat. Neurosci. 21, 1728–1741. doi: 10.1038/s41593-018-0262-6 30382196 PMC6268129

[B45] Freyvert Y. Yong N. A. Morikawa E. Zdunowski S. Sarino M. E. Gerasimenko Y. . (2018). Engaging cervical spinal circuitry with non-invasive spinal stimulation and buspirone to restore hand function in chronic motor complete patients. Sci. Rep. 8, 1–10. doi: 10.1038/s41598-018-33123-5 30341390 PMC6195617

[B46] Friederich A. R. Bao X. Triolo R. J. Audu M. L. (2022). Feedback control of upright seating with functional neuromuscular stimulation during a reaching task after spinal cord injury: a feasibility study. J. Neuroeng. Rehabil. 19, 1–13. doi: 10.1186/s12984-022-01113-4 36510259 PMC9746096

[B47] Gandolla M. Antonietti A. Longatelli V. Pedrocchi A. (2020). The effectiveness of wearable upper limb assistive devices in degenerative neuromuscular diseases: a systematic review and meta-analysis. Front. Bioeng. Biotechnol. 7, 1–16. doi: 10.3389/fbioe.2019.00450 32039171 PMC6992540

[B48] Gil-Castillo J. Alnajjar F. Koutsou A. Torricelli D. Moreno J. C. (2020). Advances in neuroprosthetic management of foot drop: a review. J. Neuroeng. Rehabil. 17, 1–19. doi: 10.1186/s12984-020-00668-4 32213196 PMC7093967

[B49] Gobbo M. Maffiuletti N. A. Orizio C. Minetto M. A. (2014). Muscle motor point identification is essential for optimizing neuromuscular electrical stimulation use. J. Neuroeng. Rehabil. 11, 1–6. doi: 10.1186/1743-0003-11-17 24568180 PMC3938308

[B50] Graczyk E. L. Resnik L. Schiefer M. A. Schmitt M. S. Tyler D. J. (2018). Home use of a neural-connected sensory prosthesis provides the functional and psychosocial experience of having a hand again. Sci. Rep. 8, 1–17. doi: 10.1038/s41598-018-26952-x 29959334 PMC6026118

[B51] Grant V. M. Gibson A. Shields N. (2017). Somatosensory stimulation to improve hand and upper limb function after stroke—a systematic review with meta-analyses. Top. Stroke Rehabil. 25, 150–160. doi: 10.1080/10749357.2017.1389054 29050540

[B52] Gull M. A. Bai S. Bak T. (2020). A review on design of upper limb exoskeletons. Robotics 9, 1–35. doi: 10.3390/robotics9010016

[B53] Harnett A. Rice D. McIntyre A. Mehta S. Iruthayarajah J. Benton B. . (2020). Upper Limb Rehabilitation Following Spinal Cord Injury. Technical report, SCIRE. Available online at: https://scireproject.com/wp-content/uploads/2022/01/upper-limb_V7.pdf (Accessed March 06, 2025).

[B54] Hatem S. M. Saussez G. della Faille M. Prist V. Zhang X. Dispa D. Bleyenheuft Y. (2016). Rehabilitation of motor function after stroke: a multiple systematic review focused on techniques to stimulate upper extremity recovery. Front. Hum. Neurosci. 10:442. doi: 10.3389/fnhum.2016.00442 27679565 PMC5020059

[B55] Höhler C. Trigili E. Astarita D. Hermsdörfer J. Jahn K. Krewer C. (2024). The efficacy of hybrid neuroprostheses in the rehabilitation of upper limb impairment after stroke, a narrative and systematic review with a meta-analysis. Artif. Organs 48, 232–253. doi: 10.1111/aor.14618 37548237

[B56] Höller Y. Tadzic A. Thomschewski A. C. Höller P. Leis S. Tomasi S. O. . (2017). Factors affecting volume changes of the somatosensory cortex in patients with spinal cord injury: to be considered for future neuroprosthetic design. Front. Neurol. 8:662. doi: 10.3389/fneur.2017.00662 29321758 PMC5732216

[B57] Hoppe-Ludwig S. Armitage J. Turner K. L. O'Brien M. K. Mummidisetty C. K. Koch L. M. . (2021). Usability, functionality, and efficacy of a custom myoelectric elbow-wrist-hand orthosis to assist elbow function in individuals with stroke. J. Rehabil. Assist. Technol. Eng. 8:205566832110350. doi: 10.1177/20556683211035057 34471545 PMC8404626

[B58] Humphreys L. Taylor P. Kwan Y.-t. (2011). Upper Limb Neuromuscular Electrical Stimulation: Electrode Positions. The National Clinical FES Centre, Salisbury District Hospital, Salisbury, Wiltshire Report. Odstock Medical Ltd. Available online at: https://odstockmedical.com/wp-content/uploads/ul_electrode_placement_handout.pdf (Accessed January 9, 2025).

[B59] Ibitoye M. O. Hamzaid N. A. Hasnan N. Wahab A. K. A. Davis G. M. (2016). Strategies for rapid muscle fatigue reduction during FES exercise in individuals with spinal cord injury: a systematic review. PLoS ONE 11:e0149024. doi: 10.1371/journal.pone.0149024 26859296 PMC4747522

[B60] Inanici F. Brighton L. N. Samejima S. Hofstetter C. P. Moritz C. T. (2021). Transcutaneous spinal cord stimulation restores hand and arm function after spinal cord injury. IEEE Trans. Neural Syst. Rehabil. Eng. 29, 310–319. doi: 10.1109/TNSRE.2021.3049133 33400652

[B61] Inanici F. Samejima S. Gad P. Edgerton V. R. Hofstetter C. P. Moritz C. T. (2018). Transcutaneous electrical spinal stimulation promotes long-term recovery of upper extremity function in chronic tetraplegia. IEEE Trans. Neural Syst. Rehabil. Eng. 26, 1272–1278. doi: 10.1109/TNSRE.2018.2834339 29877852 PMC6986544

[B62] Jabban L. Metcalfe B. W. Raines J. Zhang D. Ainsworth B. (2022). Experience of adults with upper-limb difference and their views on sensory feedback for prostheses: a mixed methods study. J. Neuroeng. Rehabil. 19, 1–18. doi: 10.1186/s12984-022-01054-y 35870940 PMC9308922

[B63] Jafari E. Descollonges M. Deley G. Di Marco J. Metani L. Popovic-Maneski A. (2025a). Comfort, consistency, and efficiency of garments with textile electrodes versus hydrogel electrodes for neuromuscular electrical stimulation in a randomized crossover trial. Sci. Rep. 15:6869. doi: 10.1038/s41598-025-91452-8 40011562 PMC11865617

[B64] Jafari E. Kajganic P. Bergeron V. Di Marco J. Metani A. Popovic-Maneski L. (2025b). Efficacy of high- versus moderate-intensity spatially distributed sequential stimulation in subjects with spinal cord injury: an isometric study. J. Neuroeng. Rehabil. 22:65. doi: 10.1186/s12984-025-01567-2 40122808 PMC11931884

[B65] Khan M. A. Fares H. Ghayvat H. Brunner I. C. Puthusserypady S. Razavi B. . (2023). A systematic review on functional electrical stimulation based rehabilitation systems for upper limb post-stroke recovery. Front. Neurol. 14:1272992. doi: 10.3389/fneur.2023.1272992 38145118 PMC10739305

[B66] Klakowicz P. M. Baldwin E. R. Collins D. F. (2006). Contribution of M-waves and H-reflexes to contractions evoked by tetanic nerve stimulation in humans. J. Neurophysiol. 96, 1293–1302. doi: 10.1152/jn.00765.2005 16611843

[B67] Kobbelgaard F. V. Kanstrup A. M. Andreasen Struijk L. N. (2021). Exploring User Requirements for an Exoskeleton Arm Insights from a User-Centered Study with People Living with Severe Paralysis, Vol. 12932. Cham: Springer International Publishing. doi: 10.1007/978-3-030-85623-6_19

[B68] Koutsou A. D. Moreno J. C. Del Ama A. J. Rocon E. Pons J. L. (2016). Advances in selective activation of muscles for non-invasive motor neuroprostheses. J. Neuroeng. Rehabil. 13:56. doi: 10.1186/s12984-016-0165-2 27296478 PMC4907085

[B69] Lauer R. T. Kilgore K. L. Peckham P. H. Bhadra N. Keith M. W. (1999). The function of the finger intrinsic muscles in response to electrical stimulation. IEEE Trans. Rehabil. Eng. 7, 19–26. doi: 10.1109/86.750547 10188604

[B70] Lawrence E. S. Coshall C. Dundas R. Stewart J. Rudd A. G. Howard R. . (2001). Estimates of the prevalence of acute stroke impairments and disability in a multiethnic population. Stroke 32, 1279–1284. doi: 10.1161/01.STR.32.6.1279 11387487

[B71] Losanno E. Mender M. Chestek C. Shokur S. Micera S. (2023). Neurotechnologies to restore hand functions. Nat. Rev. Bioeng. 1, 390–407. doi: 10.1038/s44222-023-00054-4

[B72] Mahan E. E. Oh J. Chase E. D. Dunkelberger N. B. King S. T. Sayenko D. . (2024). Assessing the effect of cervical transcutaneous spinal stimulation with an upper limb robotic exoskeleton and surface electromyography. IEEE Trans. Neural Syst. Rehabil. Eng. 32, 2883–2892. doi: 10.1109/TNSRE.2024.3436583 39088505

[B73] Malešević N. Popović Maneski L. Ilić V. Jorgovanović N. Bijelić G. Keller T. . (2012). A multi-pad electrode based functional electrical stimulation system for restoration of grasp. J. Neuroeng. Rehabil. 9:66. doi: 10.1186/1743-0003-9-66 23009589 PMC3547757

[B74] Malešević N. Popović L. Bijelić G. Kvaščev G. (2010). Muscle twitch responses for shaping the multi-pad electrode for functional electrical stimulation. J. Automat. Contr. 20, 53–58. doi: 10.2298/JAC1001053M

[B75] Manson G. A. Calvert J. S. Ling J. Tychhon B. Ali A. Sayenko D. G. . (2020). The relationship between maximum tolerance and motor activation during transcutaneous spinal stimulation is unaffected by the carrier frequency or vibration. Physiol. Rep. 8, 1–10. doi: 10.14814/phy2.14397 32170844 PMC7070156

[B76] Marquez-Chin C. Popovic M. R. (2020). Functional electrical stimulation therapy for restoration of motor function after spinal cord injury and stroke: a review. Biomed. Eng. Online 19, 1–25. doi: 10.1186/s12938-020-00773-4 32448143 PMC7245767

[B77] Mayr W. Bersch-Porada I. (2024). “Functional electrical stimulation of denervated muscles,” in Techniques and Technologies in Electrical Stimulation for Neuromuscular Rehabilitation, chapter 8, eds. I. Swain, J. Burridge, and T. Street (The Institution of Engineering and Technology), 233–256. doi: 10.1049/PBHE062E_ch8

[B78] Megía García A. Serrano-Muñoz D. Taylor J. Avendaño-Coy J. Gómez-Soriano J. (2020). Transcutaneous spinal cord stimulation and motor rehabilitation in spinal cord injury: a systematic review. Neurorehabil. Neural Repair 34, 3–12. doi: 10.1177/1545968319893298 31858871

[B79] Meyer J. T. Gassert R. Lambercy O. (2021). An analysis of usability evaluation practices and contexts of use in wearable robotics. J. Neuroeng. Rehabil. 18, 1–15. doi: 10.1186/s12984-021-00963-8 34886902 PMC8656061

[B80] Milosevic M. Marquez-Chin C. Masani K. Hirata M. Nomura T. Popovic M. R. . (2020). Why brain-controlled neuroprosthetics matter: mechanisms underlying electrical stimulation of muscles and nerves in rehabilitation. Biomed. Eng. Online 19, 1–30. doi: 10.1186/s12938-020-00824-w 33148270 PMC7641791

[B81] Milosevic M. Masugi Y. Sasaki A. Sayenko D. G. Nakazawa K. (2019). On the reflex mechanisms of cervical transcutaneous spinal cord stimulation in human subjects. J. Neurophysiol. 121, 1672–1679. doi: 10.1152/jn.00802.2018 30840527

[B82] Moineau B. Marquez-Chin C. Alizadeh-Meghrazi M. Popovic M. R. (2019). Garments for functional electrical stimulation: design and proofs of concept. J. Rehabil. Assist. Technol. Eng. 6. doi: 10.1177/2055668319854340 35186317 PMC8855467

[B83] Nakagawa K. Bergquist A. J. Yamashita T. Yoshida T. Masani K. (2020). Motor point stimulation primarily activates motor nerve. Neurosci. Lett. 736:135246. doi: 10.1016/j.neulet.2020.135246 32673689

[B84] Nakagawa K. Fok K. L. Masani K. (2023). Neuromuscular recruitment pattern in motor point stimulation. Artif. Organs 47, 537–546. doi: 10.1111/aor.14445 36305730

[B85] Nathan R. H. (1979). Functional electrical stimulation of the upper limb: charting the forearm surface. Med. Biol. Eng. Comput. 17, 729–736. doi: 10.1007/BF02441554 317915

[B86] Niu C. M. Bao Y. Zhuang C. Li S. Wang T. Cui L. . (2019). Synergy-based FES for post-stroke rehabilitation of upper-limb motor functions. IEEE Trans. Neural Syst. Rehabil. Eng. 27, 256–264. doi: 10.1109/TNSRE.2019.2891004 30763238

[B87] Ong Sio L. C. Hom B. Garg S. Abd-Elsayed A. (2023). Mechanism of action of peripheral nerve stimulation for chronic pain: a narrative review. Int. J. Mol. Sci. 24:4540. doi: 10.3390/ijms24054540 36901970 PMC10003676

[B88] Osborn L. Fifer M. Moran C. Betthauser J. Armiger R. Kaliki R. . (2018). “Targeted transcutaneous electrical nerve stimulation for phantom limb sensory feedback,” *2017 IEEE Biomedical Circuits and Systems Conference (BioCAS)* (Turin: IEEE), 1–4. doi: 10.1109/BIOCAS.2017.8325200 PMC8068407 33899051

[B89] Pan L. Ren Z. Zhu K. Li J. (2023). Eliciting tactile sensations in the hand through non-invasive proximal nerve stimulation: a feasibility study. Med. Biol. Eng. Comput. 61, 3225–3232. doi: 10.1007/s11517-023-02923-x 37721698

[B90] Patel A. Berdunov V. Quayyum Z. King D. Knapp M. Wittenberg R. . (2020). Estimated societal costs of stroke in the UK based on a discrete event simulation. Age Ageing 49, 270–276. doi: 10.1093/ageing/afz162 31846500 PMC7047817

[B91] Peckham P. H. Knutson J. S. (2005). Functional electrical stimulation for neuromuscular applications. Annu. Rev. Biomed. Eng. 7, 327–360. doi: 10.1146/annurev.bioeng.6.040803.140103 16004574

[B92] Pedrocchi A. Ferrante S. Ambrosini E. Gandolla M. Casellato C. Schauer T. . (2013). MUNDUS project: multimodal neuroprosthesis for daily upper limb support. J. Neuroeng. Rehabil. 10, 1–20. doi: 10.1186/1743-0003-10-66 23822118 PMC3733825

[B93] Phadke C. P. Robertson C. T. Condliffe E. G. Patten C. (2012). Upper-extremity H-reflex measurement post-stroke: reliability and inter-limb differences. Clin. Neurophysiol. 123, 1606–1615. doi: 10.1016/j.clinph.2011.12.012 22277759

[B94] Pollock A. Farmer S. E. Brady M. C. Langhorne P. Mead G. E. Mehrholz J. . (2014). Interventions for improving upper limb function after stroke. Cochrane Database Syst. Rev. 2014:CD010820. doi: 10.1002/14651858.CD010820.pub2 25387001 PMC6469541

[B95] Popović Maneski L. Topalović I. Jovičić N. Dedijer S. Konstantinović L. Popović D. B. (2016). Stimulation map for control of functional grasp based on multi-channel EMG recordings. Med. Eng. Phys. 38, 1251–1259. doi: 10.1016/j.medengphy.2016.06.004 27353335

[B96] Popović Maneski L. Z. Malešević N. M. Savić A. M. Keller T. Popović D. B. (2013). Surface-distributed low-frequency asynchronous stimulation delays fatigue of stimulated muscles. Muscle Nerve 48, 930–937. doi: 10.1002/mus.23840 23512421

[B97] Qu H. Xie Y. Liu X. He X. Hao M. Bao Y. . (2016). Development of network-based multichannel neuromuscular electrical stimulation system for stroke rehabilitation. J. Rehabil. Res. Dev. 53, 263–278. doi: 10.1682/JRRD.2014.10.0227 27149687

[B98] Rath M. Vette A. H. Ramasubramaniam S. Li K. Burdick J. Edgerton V. R. . (2018). Trunk stability enabled by noninvasive spinal electrical stimulation after spinal cord injury. J. Neurotrauma 35, 2540–2553. doi: 10.1089/neu.2017.5584 29786465 PMC6205803

[B99] Readioff R. Siddiqui Z. K. Stewart C. Fulbrook L. O'Connor R. J. Chadwick E. K. . (2021). Use and evaluation of assistive technologies for upper limb function in tetraplegia. J. Spinal Cord Med. 45, 809–820. doi: 10.1080/10790268.2021.1878342 33606599 PMC9662059

[B100] Resquín F. Cuesta Gómez A. Gonzalez-Vargas J. Brunetti F. Torricelli D. Molina Rueda F. . (2016). Hybrid robotic systems for upper limb rehabilitation after stroke: a review. Med. Eng. Phys. 38, 1279–1288. doi: 10.1016/j.medengphy.2016.09.001 27692878

[B101] Romero-Sánchez F. Bermejo-García J. Barrios-Muriel J. Alonso F. J. (2019). Design of the cooperative actuation in hybrid orthoses: a theoretical approach based on muscle models. Front. Neurorobot. 13:58. doi: 10.3389/fnbot.2019.00058 31417390 PMC6684761

[B102] Rossi-Durand C. Jones K. E. Adams S. Bawa P. (1999). Comparison of the depression of H-reflexes following previous activation in upper and lower limb muscles in human subjects. Exp. Brain Res. 126, 117–127. doi: 10.1007/s002210050721 10333012

[B103] Routledge N. Jabban L. Donnelly T. Zhang D. Metcalfe B. W. (2024). “Anodic transcutaneous spinal cord stimulation for eliciting upper limb motion: an alternative hypothesis,” *in Abstracts from the IFESS 2023 Conferences, Vol. 48*, e19–e20.

[B104] Rushton D. N. (1997). Functional electrical stimulation. Physiol. Meas. 18, 241–275. doi: 10.1088/0967-3334/18/4/001 9413861

[B105] Safdarian M. Trinka E. Rahimi-Movaghar V. Thomschewski A. Aali A. Abady G. G. . (2023). Global, regional, and national burden of spinal cord injury, 1990–2019: a systematic analysis for the Global Burden of Disease Study 2019. Lancet Neurol. 22, 1026–1047. doi: 10.1016/S1474-4422(23)00287-9 37863591 PMC10584692

[B106] Santello M. Baud-Bovy G. Jörntell H. (2013). Neural bases of hand synergies. Front. Comput. Neurosci. 7, 1–15. doi: 10.3389/fncom.2013.00023 23579545 PMC3619124

[B107] Sarac M. Solazzi M. Frisoli A. (2019). Design requirements of generic hand exoskeletons and survey of hand exoskeletons for rehabilitation, assistive, or haptic use. IEEE Trans. Haptics 12, 400–413. doi: 10.1109/TOH.2019.2924881 31251193

[B108] Schmid U. D. Walker G. Hess C. W. Schmid J. (1990). Magnetic and electrical stimulation of cervical motor roots: technique, site and mechanisms of excitation. J. Neurol. Neurosurg. Psychiatry 53, 770–777. doi: 10.1136/jnnp.53.9.770 2174077 PMC1014255

[B109] Shin H. Chen R. Hu X. (2018). Delayed fatigue in finger flexion forces through transcutaneous nerve stimulation. J. Neural Eng. 15:066005. doi: 10.1088/1741-2552/aadd1b 30150485

[B110] Shin H. Hawari M. A. Hu X. (2021). Activation of superficial and deep finger flexors through transcutaneous nerve stimulation. IEEE J. Biomed. Health Inf. 25, 2575–2582. doi: 10.1109/JBHI.2020.3041669 33259310

[B111] Shin H. Watkins Z. Hu X. (2017). Exploration of hand grasp patterns elicitable through non-invasive proximal nerve stimulation. Sci. Rep. 7, 1–8. doi: 10.1038/s41598-017-16824-1 29185474 PMC5707381

[B112] Smaby N. Johanson M. E. Baker B. Kenney D. E. Murray W. M. Hentz V. R. . (2004). Identification of key pinch forces required to complete functional tasks. J. Rehabil. Res. Dev. 41, 215–223. doi: 10.1682/JRRD.2004.02.0215 15558375

[B113] Snell R. S. (2012). The Upper Limb, chapter 9, 9th Edn. Philadelphia, PA: Lippincott Williams & Wilkins, 382–397.

[B114] Sousa A. S. Moreira J. Silva C. Mesquita I. Macedo R. Silva A. . (2022). Usability of functional electrical stimulation in upper limb rehabilitation in post-stroke patients: a narrative review. Sensors 22:1409. doi: 10.3390/s22041409 35214311 PMC8963083

[B115] Stephan K. Huber S. Häberle S. Kanz K. G. Bühren V. Van Griensven M. . (2015). Spinal cord injury - incidence, prognosis, and outcome: an analysis of the TraumaRegister DGU. Spine J. 15, 1994–2001. doi: 10.1016/j.spinee.2015.04.041 25939671

[B116] Stewart A. M. Pretty C. G. Adams M. Chen X. Q. (2017). Review of upper limb hybrid exoskeletons. IFAC-PapersOnLine 50, 15169–15178. doi: 10.1016/j.ifacol.2017.08.2266

[B117] Stewart A. M. Pretty C. G. Chen X. (2019). An investigation into the effect of electrode type and stimulation parameters on FES-induced dynamic movement in the presence of muscle fatigue for a voltage-controlled stimulator. IFAC J. Syst. Control 8:100043. doi: 10.1016/j.ifacsc.2019.100043

[B118] Stewart C. M. Qadri M. Y. J. Daly C. A. (2023). Upper-extremity peripheral nerve stimulators. J. Hand Surg. Glob. Online 5, 121–125. doi: 10.1016/j.jhsg.2021.12.012 36704375 PMC9870788

[B119] Tashiro S. Mizuno K. Kawakami M. Takahashi O. Nakamura T. Suda M. . (2019). Neuromuscular electrical stimulation-enhanced rehabilitation is associated with not only motor but also somatosensory cortical plasticity in chronic stroke patients: an interventional study. Ther. Adv. Chronic Dis. 10, 1–13. doi: 10.1177/2040622319889259 31798821 PMC6868577

[B120] Taylor C. McHugh C. Mockler D. Minogue C. Reilly R. B. Fleming N. . (2021). Transcutaneous spinal cord stimulation and motor responses in individuals with spinal cord injury: a methodological review. PLoS ONE 16:e0260166. doi: 10.1371/journal.pone.0260166 34793572 PMC8601579

[B121] Taylor P. Humphreys L. Swain I. (2013). The long-term cost-effectiveness of the use of functional electrical stimulation for the correction of dropped foot due to upper motor neuron lesion. J. Rehabil. Med. 45, 154–160. doi: 10.2340/16501977-1090 23303521

[B122] Tefertiller C. Bartelt P. Stobelaar M. Charlifue S. Sevigny M. Vande Griend E. . (2022). Improving upper extremity strength, function, and trunk stability using wide-pulse functional electrical stimulation in combination with functional task-specific practice. Top. Spinal Cord Inj. Rehabil. 28, 139–152. doi: 10.46292/sci21-00004 35521056 PMC9009203

[B123] Tharu N. S. Wong A. Y. L. Zheng Y. P. (2023). Neuromodulation for recovery of trunk and sitting functions following spinal cord injury: a comprehensive review of the literature. Bioelectron. Med. 9:11. doi: 10.1186/s42234-023-00113-6 37246214 PMC10226194

[B124] Tigra W. Dali M. William L. Fattal C. Gélis A. Divoux J. L. . (2020). Selective neural electrical stimulation restores hand and forearm movements in individuals with complete tetraplegia. J. Neuroeng. Rehabil. 17, 1–12. doi: 10.1186/s12984-020-00676-4 32429963 PMC7236876

[B125] Tresilian J. (2012). Sensorimotor Control and Learning: An Introduction to the Behavioral Neuroscience of Action, Chapter 1. Basingstoke: Palgrave Macmillan, 3–37.

[B126] Trigili E. Grazi L. Crea S. Accogli A. Carpaneto J. Micera S. . (2019). Detection of movement onset using EMG signals for upper-limb exoskeletons in reaching tasks. J. Neuroeng. Rehabil. 16, 1–16. doi: 10.1186/s12984-019-0512-1 30922326 PMC6440169

[B127] Triolo R. Nathan R. (1996). Challenges to clinical deployment of upper limb neuroprostheses. J. Rehabil. Res. Dev. 33:111. 8724167

[B128] United Nations Department of Economic and Social Affairs, Population Division. (2024). World Population Prospects 2024. Available online at: https://population.un.org/wpp/graphs (Accessed September 4, 2025).

[B129] Usman H. Zhou Y. Metcalfe B. Zhang D. (2020). A functional electrical stimulation system of high-density electrodes with auto-calibration for optimal selectivity. IEEE Sens. J. 20, 8833–8843. doi: 10.1109/JSEN.2020.2983004

[B130] Vidaurre C. Irastorza-Landa N. Sarasola-Sanz A. Insausti-Delgado A. Ray A. M. Bibián C. . (2023). Challenges of neural interfaces for stroke motor rehabilitation. Front. Hum. Neurosci. 17:1070404. doi: 10.3389/fnhum.2023.1070404 37789905 PMC10543821

[B131] Waddell K. J. Birkenmeier R. L. Bland M. D. Lang C. E. (2016). An exploratory analysis of the self-reported goals of individuals with chronic upper-extremity paresis following stroke. Disabil. Rehabil. 38, 853–857. doi: 10.3109/09638288.2015.1062926 26146964 PMC4809414

[B132] Waltz J. M. Andreesen W. H. Hunt D. P. (1987). Spinal Cord stimulation and motor disorders. Pacing Clin. Electrophysiol. 10, 180–204. doi: 10.1111/j.1540-8159.1987.tb05947.x 2436177

[B133] Wang Y. Metcalfe B. Zhao Y. Zhang D. (2020). An assistive system for upper limb motion combining functional electrical stimulation and robotic exoskeleton. IEEE Trans. Med. Robot. Bionics 2, 260–268. doi: 10.1109/TMRB.2020.2990318

[B134] Wiest M. J. Bergquist A. J. Heffernan M. G. Popovic M. Masani K. (2019). Fatigue and discomfort during spatially distributed sequential stimulation of tibialis anterior. IEEE Trans. Neural Syst. Rehabil. Eng. 27, 1566–1573. doi: 10.1109/TNSRE.2019.2923117 31265401

[B135] Wolf D. Dunkelberger N. McDonald C. G. Rudy K. Beck C. O'Malley M. K. . (2017). “Combining functional electrical stimulation and a powered exoskeleton to control elbow flexion,” in 2017 International Symposium on Wearable Robotics and Rehabilitation, WeRob 2017 (Houston, TX), 1–2. doi: 10.1109/WEROB.2017.8383860

[B136] World Health Organization (2011). World Report on Disability. Geneva: World Health Organization, The World Bank.

[B137] World Health Organization (2021). Ageing and Health. Geneva: World Health Organization.

[B138] Yan T. Fortune B. C. Liu L. Liu Y. Kaiju T. Suzuki T. . (2025). Epineural stimulation on distal brachial plexus for functional restoration of the upper limb in a primate study. Front. Neurol. 16:1515986. doi: 10.3389/fneur.2025.1515986 40170903 PMC11958176

[B139] Yang K. Freeman C. Torah R. Beeby S. Tudor J. (2014). Screen printed fabric electrode array for wearable functional electrical stimulation. *Sens. Actuat*. Phys. 213, 108–115. doi: 10.1016/j.sna.2014.03.025

[B140] Zheng Y. Hu X. (2019). Elicited finger and wrist extension through transcutaneous radial nerve stimulation. IEEE Trans. Neural Syst. Rehabil. Eng. 27, 1875–1882. doi: 10.1109/TNSRE.2019.2930669 31352346

[B141] Zheng Y. Hu X. (2020a). Elicited upper limb motions through transcutaneous cervical spinal cord stimulation. J. Neural Eng. 17:036001. doi: 10.1088/1741-2552/ab8f6f 32357351

[B142] Zheng Y. Hu X. (2020b). Muscle activation pattern elicited through transcutaneous stimulation near the cervical spinal cord. J. Neural Eng. 17:016064. doi: 10.1088/1741-2552/ab5e09 31791027

